# The Global Environmental Benefits of Halving Avoidable Consumer Food Waste

**DOI:** 10.1021/acs.est.4c04140

**Published:** 2024-07-29

**Authors:** Antoine Coudard, Zhongxiao Sun, Paul Behrens, José Manuel Mogollón

**Affiliations:** † Institute of Environmental Sciences (CML), Leiden University, PO Box 9518, Leiden 2300 RA, The Netherlands; ‡ Metabolic Institute, Klimopweg 150, Amsterdam 1032 HX, The Netherlands; § College of Land Science and Technology, China Agriculture University, Beijing 100193, China

**Keywords:** consumer food waste, MRIO, sustainable food system, embedded environmental impacts, SDG 12.3.b

## Abstract

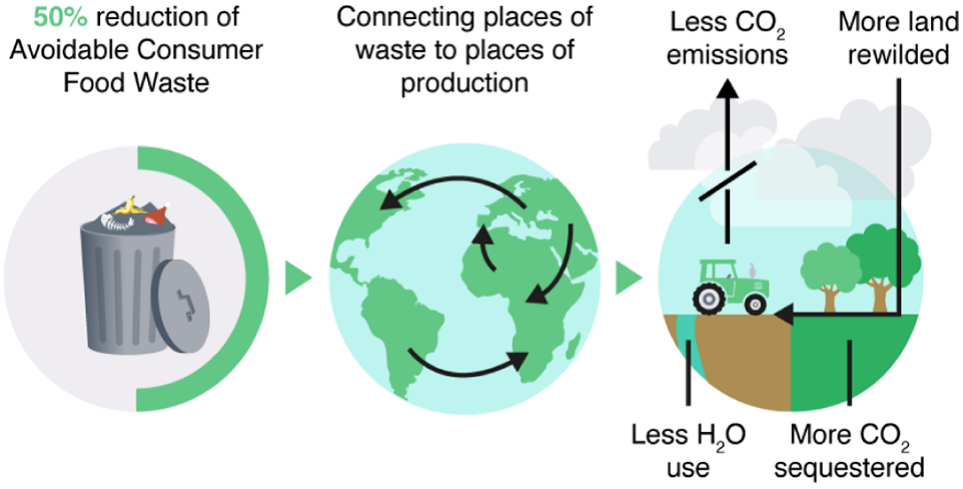

Avoidable consumer food waste (ACFW) is a global environmental issue wasting key resources and causing emissions, especially in high food-producing nations. We trace ACFW to its origin to assess emissions, water use, and land use. We show that ACFW impacts are dominated by commodities like beef, dairy, rice, and wheat. Over 80% of impacts are domestic, but impacts embodied in trade affect a few major food-producing countries under environmental pressure. A 50% reduction in ACFW could save up to 198 Mt CO2eq in emissions, 30 Gm^3^ of blue water, and 99 Mha of land. Targeting key commodities in impactful countries (e.g., US beef waste) could achieve significant benefits. Sparing wasted land and returning it to its potential natural vegetation could sequester 26 Gt CO_2_eq long-term (17–35 Gt CO_2_eq). Finally, while the 50% ACFW reduction lines up with Sustainable Development Goal (SDG) 12.3b for the avoidable portion of food waste, a total of 276 Mt of unavoidable consumer food waste is also generated, which cannot be readily reduced. Achieving a 50% reduction in total food waste would require a 93% reduction in ACFW. Tracking the spatial impacts of ACFW can elucidate the concrete benefits of policies aiming at SDG 12.3b.

## Abstract 2

Global avoidable consumer food waste squanders land and resources while causing significant greenhouse gas emissions. Halving this waste can free up 99 Mha and sequester 26 GtCO_2_eq.

## Introduction

Food systems represent a major driver of environmental impacts, yet over 1.3 Gt yr^–1^ of food products are wasted across the global food supply chain.^1^ Food loss and waste (FLW) have emerged as critical global challenges with multifaceted impacts on environmental sustainability, human health, and food security. Recent studies have highlighted FLW’s substantial contribution to greenhouse gas emissions,^2,3^ as well as its broader effects on human health (e.g., air pollution), ecosystem resilience (e.g., biodiversity),^4^ and emerging nutritional pressures.^5^ Food waste comprises both avoidable and unavoidable forms. Unavoidable food waste represents the inedible parts of food products, such as shells and peels. Avoidable food waste constitutes nonconsumed edible food, mostly from households and food services, such as restaurants. Avoidable food waste at the consumption stage constitutes a quarter of total food waste and loss globally.^6^

Sustainable Development Goal (SDG) subindicator 12.3.b has set a target of halving global food waste at the retail and consumer levels by 2030.^7^ Halfway through the 2015–2030 SDG period, only a handful of countries, representing around 35% of the global population, have drafted policies to meet this target,^8,9^ and food waste may double by 2050.^10^ Only limited exploration has been conducted on the environmental gains and land use opportunities that arise from reducing avoidable consumer food waste (ACFW) to meet the SDG 12.3 target. Furthermore, while some research has explored reducing environmental pressures by modeling reductions in FLW to meet the SDG 12.3 target on specific countries,^11^ few studies have provided a comprehensive analysis focused on ACFW. Such an analysis is required to elucidate the spatial dynamics of its environmental impacts and pressures across production and waste countries.

Another key knowledge gap in current research revolves around assessing the achievability of SDG 12.3, particularly considering both avoidable and unavoidable food waste. Despite recognition of the significance of SDG 12.3 in reducing food waste, there is limited understanding of how accounting for both types of waste influences the feasibility of achieving this goal.

Here, we combine global food trade models with a consumer food waste database to assess the domestic and trade-related environmental impacts of ACFW for 2010. We provide a country-level analysis of the environmental impacts of wasted land and water resources and GHG emissions, occurring both domestically and abroad through trade. We discuss the environmental benefits of achieving the SDG 12.3.b target at the national level for ACFW. We specifically explore the rewilding potential of resulting freed-up land and its carbon sequestration opportunities, alongside a reduction in greenhouse gas emissions and resource consumption from avoided production. Finally, we provide perspectives on the feasibility of achieving the SDG 12.3 target based on its current definition and showcase how targeted policies can fast-track concrete gains toward halving ACFW.

## Materials and Methods

Building on the models developed by Coudard et al.^6^ and Sun et al.,^12^ we developed a model that connects avoidable consumer food waste to production areas, allowing us to trace the localized environmental impacts of wasted food. Coudard et al.’s model enables us to calculate the quantities of avoidable and unavoidable consumer food waste for each country. Sun et al.’ model allows us to connect food commodities from their countries of consumption to their countries of production and then spatialize crops and animal production to determine their areas of production within each country. The model then enables to quantify the carbon sequestration potential of these production areas by comparing their current land use (i.e., agriculture) to their potential natural vegetation (PNV), should they be spared from agriculture production. An overview of these models and their interactions for this study is provided in the Supporting Information. After combining these models, we simulated the halving of avoidable consumer food waste across all countries and the avoidance of their original production (freeing up the arable land), quantifying both the environmental benefits from the avoided production and the avoided waste treatment process. We then quantified the carbon sequestration potential of the arable land spared by the reduced food production by simulating the return of PNV in these areas. See Supporting Information for a detailed schematic describing the entire workflow of the study (Figure S11).

### Avoidable Consumer Food Waste Model

Following Coudard et al.,^6^ we quantify the amounts of available food at the consumption-stage (households and food services) using the Food and Agriculture Organization’s statistical database (FAOSTAT^13^) and its Food Balance Sheets (FBSs) that compile the food available at the distribution stage in each country. The FBSs provide the average food supply at the national level for about 90 food product types or 18 aggregated food groups. The conversion from raw equivalent to product-weight is necessary to calculate the actual amount of avoidable food waste at the consumption stage. Technical conversion factors (TCFs) are used to correct the FBSs for every country, and the nature of food products (processed or fresh) is taken into consideration due to different food waste incidence rates (eq 1).
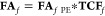
1where **FA***_f_* is the corrected, actual quantity of a food item *f* available at the Retail/Distribution stage, in kg; **FA***_f_*_PE_ is the primary-equivalent quantity of food item *f,* compiled in the FBS, in kg; and **TCF***_f_* is the technical conversion factor of food item *f,* as a percentage.

The losses at the retail-level are computed using the FAO Global Food Losses and Waste landmark report.^14^ A harmonization of food items classification is required to match the food categories used in the Global Food Losses and Waste estimates to the 18 aggregated food groups of the FBSs. This step yields the actual amounts of food that reach households and food services in each country.

The FAO Global Food Losses and Waste report provides estimates of waste percentages across various stages of the food supply chain (e.g., 5% of food waste for fruits and vegetables in Sub-Saharan Africa at the consumption stage). However, these estimates do not initially differentiate between avoidable and unavoidable, simply reporting total food waste. The report applies generic conversion factors to calculate the edible portion of the calculated food waste. This is done, however, as a very high-level of aggregation across the aggregated food waste categories (e.g., fruits and vegetables). In contrast, we collected more detailed data on the unavoidable (inedible) portions of freshly consumed products, especially for the fruits and vegetables categories but also for starchy roots, coffee, tea, seafood, and meat. In practice, we employ a more detailed “waste floor″^15^ approach to determine the minimal amounts of UCFW associated with the consumption of fresh food in households and food services. Data from various sources are used to estimate the waste fractions for different types of food such as vegetables, fruits, starchy roots,^16^ meat (bovine, pork, poultry, sheep), stimulants (coffee and tea grounds), fish and seafood,^16^ and eggs.^17^ This inedible fraction data set is applied to all countries’ food commodities. Processed food products are considered entirely edible, as the inedible portions are removed during processing. The inedible fractions of relevant food products are matched with their respective food groups, and the total amount of UCFW is calculated for each country by multiplying the fraction with the total available amounts of fresh food products made available to households and food services.

The total amounts of UCFW are calculated following eq 2.
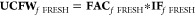
2where **UCFW***_f_* is the inedible quantity of a food item *f*, consumed fresh, that is generated at the Consumption stage, in kg; **FAC***_f_*_FRESH_ is the quantity of a food item *f*, fresh, available at the Consumption stage (food services and households), in kg; **IF***_f_*_FRESH_ is the inedible fraction of food item *f,* consumed fresh, as a percentage; **UCFW***_f_*_PROCESSED_ is considered to be 0 as the processed food item *f* is considered to have been stripped of the inedible, or unavoidable waste elements.

Avoidable consumer food waste (AFCW) is calculated by subtracting the inedible fraction for each food commodity, consumed fresh.
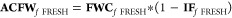
3

where **ACFW***_f_*_FRESH_ is the edible quantity of a food item *f*, consumed fresh, that is wasted at the Consumption stage, in kg. **FWC***_f_*_FRESH_ is the quantity of a food item *f*, consumed fresh, wasted at the Consumption stage, in kg. **IF***_f_*_FRESH_ is the inedible fraction of food item *f,* consumed fresh, as a percentage.

Coudard et al.^6^ and the Supporting Information provide further details the methodologies and assumptions surrounding the ACFW model.

### Connecting ACFW to Its Original Production Area

The MRIO model uses the Food and Agriculture Biomass Input–Output data set (FABIO)^18^ to link the ACFW to countries of primary agricultural production. We selected data for the year 2010 from FABIO. The data covers 191 countries and 128 agricultural, food, and forestry products from 1986 to 2013. Data on food items are then combined and harmonized with the ACFW global data set. Since it is also based on the same FAOSTAT nomenclature, the avoidable food waste items were readily matched to FABIO food items. As a result, avoidable food waste at the consumer level can be related back to the locations of their primary agricultural production.

### Harvested Land, GHG Emissions, and Blue Water from the Production of ACFW

Once the ACFW model is integrated into FABIO, we can assess harvested land, GHG emissions, and blue water consumption of various food commodities during their production.

The harvested area used to grow the avoidable food waste is quantified using FAOSTAT crop and pasture area data, combined with SPAM, a spatial production allocation model^19^ for 29 herbaceous crops, and EarthStat,^20^ a spatially explicit cropland and pastureland information data set for the fodder crops. This allows us to quantify the spatially explicit environmental impacts of food production that will become ACFW. A GHG emissions data set retrieved from Sun et al.,^12^ and originally derived from FAOSTAT at the national level, is linked to FABIO to quantify emissions from the agricultural activities that occur to produce the food items that ultimately become ACFW. The GHG emissions estimates were built using an older version of 100-year Global Warming Potentials (GWP), with those from the IPCC Fifth 5 Assessment Report (AR5) with climate-carbon feedback (that is, 34 CO_2_e for CH_4_ and 298 CO_2_e for N_2_O). The same process is performed to quantify the blue water use during the agricultural production stage of the commodities, using data sets from the Water Footprint Network^21,22^ for crop products and FAOSTAT for livestock products.^23^

Further details on the MRIO model methodologies and assumptions are in Sun et al.^12^ and Supporting Information.

### Halving Avoidable Consumer Food Waste

We use the UN SDG 12.3.b target as a basis for modeling a reduction in avoidable food waste and estimating the amounts of land that could potentially be restored to their PNV. The simplified approach halves ACFW (50% reduction) across all food categories in every country. The avoidable food waste reduction scenario is highly idealized–as it is meant to explore the potential magnitude of such a shift on global natural resources and GHG emissions. The total environmental impacts (land use, blue water, and GHG emissions) that occurred during the production of ACFW are therefore halved.

### GHG Emissions Reduction from the Avoided ACFW End-Of-Life

A GHG emissions data set derived from FAOSTAT^13^ links the total quantities of food waste generated in each country with the total GHG emissions from the waste treatment activities (e.g., landfill), in tCO_2_eq. This model^24^ determines the municipal waste treatment activities (excl. industrial waste) based on data from the *WhataWaste2.0* data set.^45^ For each country, the total food waste reaching the municipal waste treatment activities is there defined as
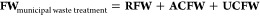
4

Where **FW**_municipal waste treatment_ is the total amount of food waste reaching municipal waste treatment activities in the given country. **RFW** is the quantity of a food waste from food retail, **ACFW** is the quantity of avoidable consumer food waste. **UCFW** is the quantity of unavoidable consumer food waste.

We then isolate the mass share of ACFW relative the total food waste reaching municipal waste treatment, for each country.
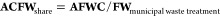
5

where ACFW_share_ is the share, in percentage mass, of avoidable consumer food waste relative to all food waste reaching municipal waste treatment activities.

We then allocate a share of the total emissions (retrieved from FAOSTAT) from municipal waste treatment for food waste for a given country to its ACFW.
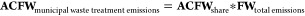
6

where ACFW_municipal waste treatment emissions_ is the GHG emissions associated with the waste treatment of ACFW in a given country. FW_total emissions_ is the total amount of GHG emissions (in tCO_2_eq) from the municipal waste treatment of food waste.

The potential GHG emissions reduction from halving ACFW in each country is then computed by halving the total emissions from ACFW of the country. While we computed the avoided production and end-of-life GHG emissions since we were particularly interested in the impacts of ACFW in places of production and waste, we did not include other sectors such as transportation, processing, wholesale, retail, hotel, and restaurant food emissions.

### PNV and Carbon Sequestration Opportunities

For the carbon sequestration benefits, we adopt Sun et al.’s^12^ approach where agricultural production is mapped using SPAM to spatially explicit cropland and pastureland, which we link to the latest harmonized global of the aboveground biomass carbon (AGBC) and belowground biomass carbon (BGBC) densities maps;^25^ a soil organic carbon (SOC) stock map of the top 100 cm;^26^ and a PNV maps with AGBC, BGBC^27^ and SOC.^28^ For both AGBC and BGBC, we allocated them into grid cells based on the spatial distribution of SPAM for crops and EarthStat for fodder crops.
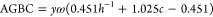
7
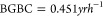
8

Where *y* is the production of a specific crop or fodder item, ω is the dry-matter fraction of its harvested biomass, *h* is its harvest index (fraction of total AGBC collected at harvest), *c* is the carbon-content fraction of its harvested dry mass, and *r* is the root-to-shoot ratio of the crop.

We determine the resulting carbon sequestration potential as the difference between the carbon stock of PNV and that of current use, following Sun et al.’s approach of a one-time “committed” mass of carbon that is sequestered over an unspecified period after restoration is initiated (in practice on the order of 40–60 years). A detailed account of the methodologies, data sets, and assumptions can be found in the Supporting Information and Sun et al. Finally, we estimated the amounts of potential carbon sequestered due to sparing 50% of the land that was dedicated to produce ACFW.

### Limitations

Blue water and GHG estimates for both the production and the waste treatment of ACFW are country-specific and are not available at the subnational level. Variations at the subnational level due to farming practices and local climate, however, are likely. This limitation provides an avenue for further research on the spatialization of blue water consumption within subnational boundaries.

Beyond the spatial granularity of data, the scope of the analysis focuses primarily on the impacts of food waste in places of production and waste. We did not include other nonagricultural sectors such as transportation, processing, and retail. which may underestimate the full life-cycle impacts of consumer food waste. The full life-cycle impacts of consumer food waste would therefore be expected to be larger than the figures presented below in this study.

## Results and Discussion

### Global Environmental Impacts of ACFW

**Figure 1:**
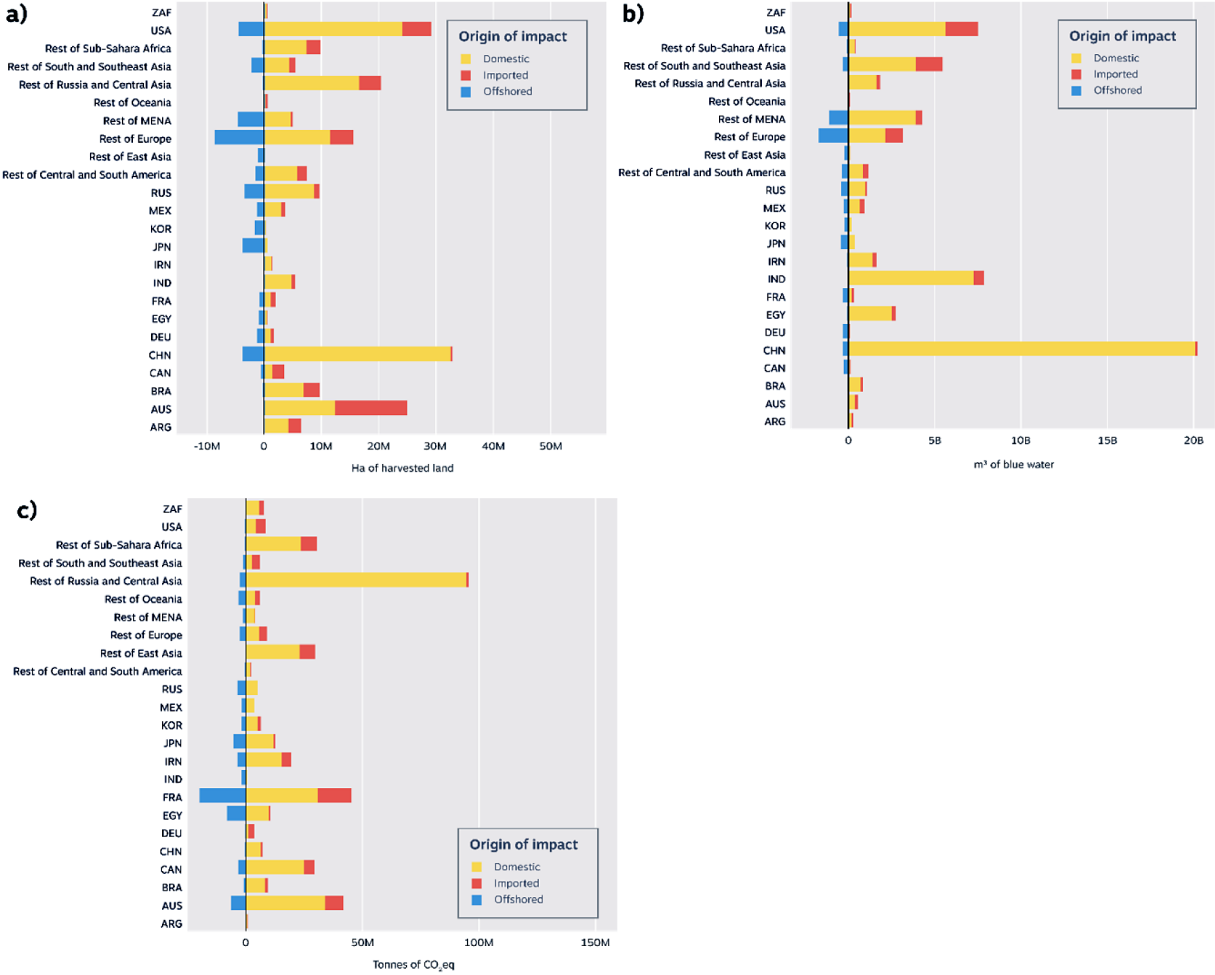
Domestic, imported, and offshored wasted (a) harvested land area (ha), (b) blue water (m^3^), and (c) GHG emissions (tCO_2_eq) from ACFW across countries and regions.

In 2010, ACFW represented an annual loss of 323 Mt, ∼ 25% of the total global food loss and waste.^6^ During its production, this food emitted 396 Mt CO_2_eq (100 GWP), almost 6% of the global agricultural (farm-gate) GHG emissions for that year.^13^ Its blue water footprint amounted to 61 billion m^3^ (Gm^3^), about ∼7% of the total blue water consumption of global agriculture.^13^ Finally, 198 Mha of land (for food crops, feed crops, and grazing), about 4% of the 4.8 Gha used for global agriculture^13^ were wasted. Land resources have a significant opportunity cost because of their potential to sequester carbon.^12^ Specifically, when restored to their PNV, these 198 Mha could result in a 52 GtCO_2_e of carbon storage. Most ACFW production impacts (82% of GHG emissions, 87% of blue water consumption, and 78% of agricultural land) occur in the same country where the waste takes place. ACFW’s environmental impacts origins can be domestic (food produced and wasted domestically), imported (food produced domestically, wasted abroad), or offshored (food produced abroad, wasted domestically), and these vary significantly throughout the world (Figure 1a–c). While a few key commodities, mainly rice, beef, and wheat, contributed to most impacts, these variations are also driven by diets, waste patterns, and trade.

Several Asian countries saw significant ACFW-related domestic environmental impacts, driven by high self-sufficiency goals regarding national grain consumption.^29^ In China, domestic ACFW represented a 6% loss of its total agricultural land.^9^ Of this loss, rice and wheat accounted for 17% and 12%, respectively (Figure S4). China’s domestic ACFW emitted 95 MtCO_2_eq (Figure 1c), with 44.5% due to rice (Figure S7), a problematic commodity for methane emissions.^30^ Meeting the SDG 12.3 target, even solely for rice products, could support China in achieving its emerging pledge on methane reduction targets.^31^ China’s ACFW required 20.4 Gm^3^ of blue water, 99% of which was from domestic water resources (Figure 1), mainly for rice (44%) and wheat (44%) (Figure S9). Alarmingly, 20% of China’s cropland and 13% of its pasture are at risk due to increasing water scarcity.^32^ In India, ∼7.2 Gm^3^ of domestic blue water was used for ACFW (Figure 1), mostly for wheat (40%) and rice (26%) (Figure S10). In North Africa, Egypt harbors large domestic blue water losses at 2.5 Gm^3^ (Figure 1), of which 74% result from domestically produced rice, wheat, and maize. As 50% of the land used to produce wheat in this region is vulnerable to water scarcity,^32^ these losses must be immediately tackled.

In the USA, the largest producer and consumer (per capita) of beef,^13^ domestic grazing land represented 73% (Figure S5) of the 24.6 Mha of land lost (Table S5). In South America, Brazil, another significant consumer of beef,^13^ emitted 24 MtCO_2_eq (Figure 1c) during the primary production of its ACFW. The vast majority (89%) stemmed from the production of domestic beef products (Figure S8). The significant quantities of GHG emissions from ACFW indicate that recent emissions targets for the agricultural sector (1.1 GtCO_2_eq reduction by 2030)^33^ set by the Brazilian government will be harder to reach without tackling ACFW. Similarly, in Oceania, 25 Mha of Australian land was wasted via ACFW, of which 93% was used for beef and sheep products (Figure S6). About 50% of these products were wasted abroad, reflecting the export-oriented nature of Australia’s beef and sheep industries.^34^

### Unevenly Traded Impacts

**Figure 2:**
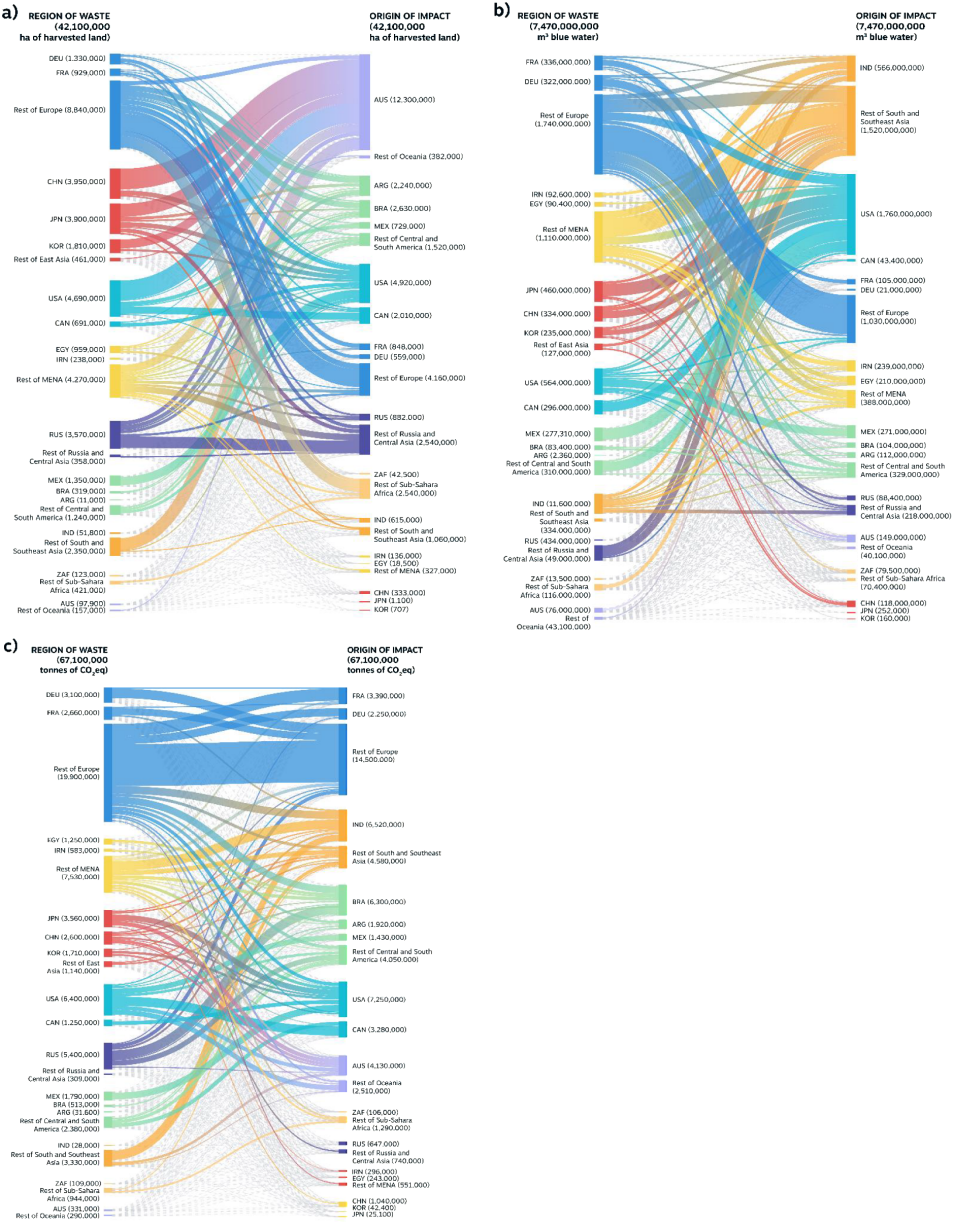
(a) Harvested land area (ha) traded between region of waste and region of production. (b) Blue water (m3) traded between regions of waste and regions of production. (c) GHG emissions (ton CO_2_eq) traded between region of waste and region of production.

In 2010, ACFW from traded commodities represented a loss of 42 Mha of land, 7.4 Gm^3^ of blue water, and 67 Mt CO_2_eq of GHG emissions, indicating the sizable, offshored impacts to food-producing nations (Figure 2a–c). European countries offshored the largest amounts of GHG (∼26 MtCO_2_eq, Figure 2c) and land impacts (∼11 Mha, 26% of all traded land impacts, Figure 2a) related to their ACFW. Although mostly trading the impacts between European countries due to their intense trading network, they still induced significant impacts beyond the continent, with 21% and 15% of these land losses located in South America and sub-Saharan Africa, respectively. East Asian nations accounted for ∼10 Mha of offshored wasted land (Table S2), with 5 Mha located in Australia, mostly for grazing. In North America, ACFW from the USA accounted for 4.7 Mha losses, more than half (2.4 Mha) originating in Australia, primarily from cattle grazing (Figure 2). The USA offshored the largest amount of GHG emissions of any nation, with 6.4 MtCO_2_eq, mostly related to beef (∼70%).

Commodity-wise, cattle and meat products vastly contribute to traded embedded impacts. Cattle-related products represent 9% of total ACFW mass, but 48% of ACFW’s embedded GHG. This agrees with studies that highlight the outsized impacts of beef compared to its mass, calorie, and protein content.^35^ This showcases those countries producing large amounts of beef products for export markets (e.g., USA, Brazil, Australia) would benefit greatly from the reduction of beef ACFW beyond their own boundaries.

Several key food-producing nations, mainly Australia, the USA, Brazil, and India, as well as Southeast Asian countries like Thailand and Pakistan, are bearing the brunt of ACFW traded impacts (Figure 2a–c). Australia, where 90% of farming land is dedicated to grazing,^36^ used 12.8 Mha for beef and sheep products that became ACFW abroad (Table S4). Its land impacts amounted to 29% of all traded land impacts. It also emitted 4.3 MtCO_2_eq during the production of this nondomestic ACFW. The USA emitted 7.7 MtCO_2_eq for edible food wasted abroad, mostly related to rice (∼26%), beef (∼22%), and wheat products (12%). In Brazil, a country experiencing land competition and biodiversity loss,^37^ 2.8 Mha of mostly soy-producing and grazing lands were lost to ACFW abroad (Table S4). Beef ACFW in Russia alone (their largest importer^38^) accounted for 1.7 MtCO_2_eq of Brazilian GHG emissions. This is the single largest country-to-country offshoring of GHG emissions (Table S14).

Pakistan, India, and Thailand, major rice producers, are already experiencing increasing water scarcity^39,40^ and lost 0.9 Gm^3^, 0.6 Gm^3^, and 0.5 Gm^3^ of freshwater resources via ACFW abroad, respectively (Table S9). This needless waste is adding pressure on Pakistan’s food system which has seen in recent years its wheat yield decrease by almost 5% due to water shortages, and this trend may worsen by 2035 for both wheat and rice production.^41^ The loss of nonrenewable freshwater resources is also concerning for the USA (1.9 Gm^3^, Table S9), which struggles with water stress^42^ and may see lower rainfall and wheat yields decrease by midcentury in some of its regions.^43^

### Environmental Benefits from Halving ACFW

#### Avoided Production Impacts

**Figure 3:**
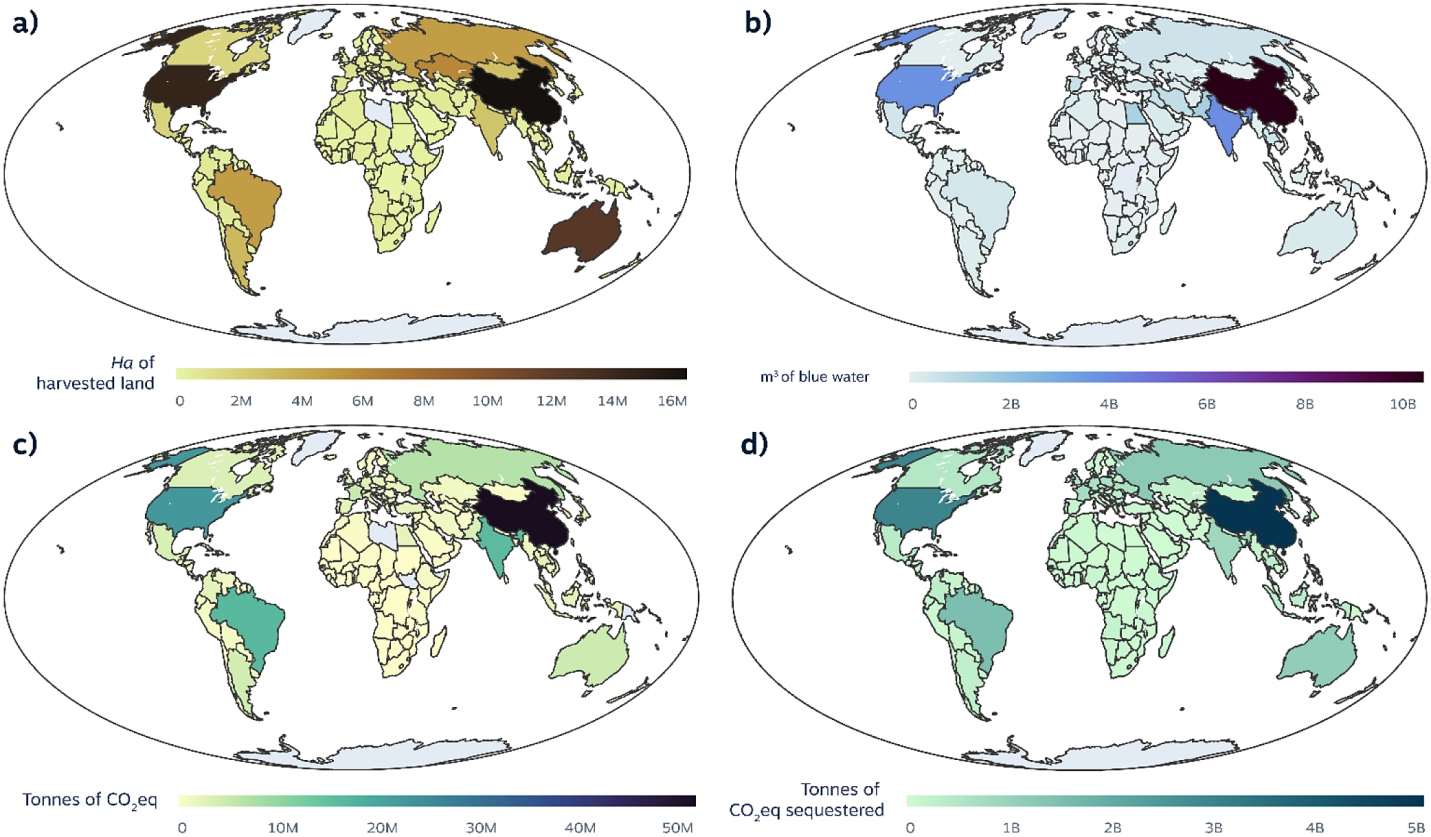
(a) Harvested land area spared (ha). (b) Production-based GHG emissions avoided (tons CO_2_eq). (c) Production-based blue water use avoided (m^3^). (d) GHG emissions stored from allowing the land to return to its PNV (tons CO_2_eq).

Halving ACFW would free 99 Mha of arable land. (Figure 3a). Freed land in China (17Mha), USA (15 Mha), and Australia (13Mha), would help alleviate the pressure these countries face in terms of increased land aridity.^44^ Further, global ACFW blue water loss would fall by 30.5 Gm^3^. Here, China (10 Gm^3^), India (3.9 Gm^3^), the USA (3.7 Gm^3^), Pakistan (1.4 Gm^3^), and Egypt (1.3 Gm^3^) would benefit most (Figure 3b). These countries are all experiencing blue water availability issues^42^ and represent 70% of the total unsustainable blue water footprint of global food production.^100^ More generally, halving ACFW globally would help improve the global water footprint of agriculture currently estimated at 57% of unsustainable blue water use.^100^ Lastly, global ACFW emissions would diminish by 198 MtCO_2_eq, equivalent to 3% of farm-gate emissions in 2010.^13^ The largest reductions in emissions would occur in China (48 MtCO_2_eq, 24% of the total savings), the USA (10.5%), Brazil (8%), and India (7.5%) (Figure 3). Countries that have been significantly impacted by offshored GHG emissions (Figure 2c), such as Brazil (∼7 MtCO_2_eq emitted for oversea ACFW), and Australia (∼4.3 MtCO_2_eq) would benefit from these reductions to meet their own climate targets.

Several commodity-nation pairs have a significant contribution to the total impacts of ACFW. For instance, a 50% ACFW reduction of Chinese rice products would account for, respectively, 11% and 15% of the total GHG and blue water savings from halving all ACFW. Halving ACFW of meat products in the USA would account for almost 11% of the total global land regained from halving all ACFW. Overall, halving the ACFW of the respective single most impactful commodity across the top five countries (e.g., China and its rice waste or the USA and its beef waste) would already achieve at least ∼25% of the total savings possible from halving all ACFW.

#### Avoided End-of-Life Treatment Impacts

The benefits of reaching this target would not be limited to food production regions. Globally, the vast majority of ACFW ends up in landfills and open dumps^45^ contributing to methane emissions. Halving ACFW would lead to an estimated reduction of 224 MtCO_2_eq (18% of the global food waste treatment emissions in 2010^13^). The greatest reductions in food waste treatment emissions would occur in China (42 MtCO_2_eq), the USA (24 MtCO_2_eq), India (15 MtCO_2_eq), and Brazil (14 MtCO_2_eq). A reduction in ACFW would also have positive impacts on water and land resources that are either used or negatively impacted by waste treatment activities. While a global understanding of the land footprint and water impacts of food waste management does not exist yet, landfills do cover significant surface areas, and leachates pollute groundwater and surface water resources.^46^

#### Carbon Sequestration Opportunities

Halving ACFW frees land for uses different from food production. Lowering food production opens the opportunity for rewilding freed-up land.^12^ Reverting 99 Mha of land from saving 50% of global AFWC toward natural vegetation would result in 26 GtCO_2_eq of carbon storage. Almost two-thirds of this carbon sequestration opportunity would be located in pastureland (40%), and arable land that mainly grows wheat (14%), and rice (9%). As one-third of the planet’s soils are degraded,^47^ halving ACFW would support the regeneration of these lands. China, the USA, Brazil, Russia, and Australia see the greatest carbon sequestration opportunities (Figure 3d), amounting to 12 GtCO_2_eq (46% of the total). These represent almost six years (2010–2016) of their combined farm-gate GHG emissions.^13^ European countries would sequester 5.3 GtCO_2_eq (22% of the total). A promising double dividend in GHGs is therefore possible by reducing food production and coupling freed lands to policies encouraging rewilding. Additional benefits from rewilding agricultural and pastureland include the regeneration of local biodiversity,^48^ and renewability improvements of blue freshwater resources.^49^

### Sensitivity Analysis

In this study, we conducted a sensitivity analysis on the food waste data used in the ACFW data set. Data on the confidence level of household food waste data set, ranging from *High confidence* to *Very low confidence,* were collected for each country from the UNEP Food Waste Index annexes. Confidence ranges were then built following the report’s suggestions by attributing each country’ confidence level to the specific confidence range advised in the report (e.g., +15% or −15% is suggested for countries with high confidence food waste estimate). The confidence ranges are then applied to the ACFW data set and subsequently integrated into the MRIO model. The sensitivity analysis results for global harvested land vary from a minimum of 127 Mha to a maximum of 270 Mha. The blue water consumption results vary from 38 Gm^3^ to 82 Gm^3^. The GHG results show production emissions varying from 254 MtCO_2_eq to 540 MtCO_2_eq while the waste treatment emissions varied from 335 MtCO_2_eq to 530 MtCO_2_eq, globally. The carbon sequestration potential from halving AFCW vary from 17 GtCO_2_eq to 35 GtCO_2_eq. See Supporting Information for a full description and results of the sensitivity analysis (Figures S12–S26).

### Policies Toward Halving ACFW

Halfway through the 2015–2030 SDG period, emerging food waste reduction policies are limited and have seen little success.^8^ For example, the USA’s food waste policies are mostly limited to liability protection for food donors and distributors.^50^ Such policies exclude households and food services, where the majority of ACFW occurs. Ambitiously, the European Union seeks a 50% legally binding reduction target for its member states in the coming years.^51^ However, concrete details beyond improved product expiry date labeling have yet to emerge. Recently, China has enacted more concrete policies to reduce the promotion of excessive food consumption by limiting leftovers at restaurants and as well as restricting social media content promoting overeating.^52^ Meeting the 50% food waste reduction target at the household-level may free up resources that consumers reallocate to nonfood expenditure, increasing direct, indirect, and economy-wide consumption.^53^ This rebound effect can limit the environmental benefits of reducing ACFW, so our result should be interpreted as a best-case scenario. Nonetheless, household food waste reduction exhibited the smallest rebound effect when compared to a reduction of food waste and loss in other stages of the supply chain (e.g., production, processing).^54^ Tackling ACFW based on its volume and more limited rebound effects remains an important cornerstone for the SDG 12.3b target.

The achievability of SDG 12.3b may also depend on the chosen definitions of food waste. The SDG 12.3b target includes both avoidable and unavoidable food waste.^7^ In 2010, 276 Mt of unavoidable consumer food waste were generated. This represents 46% of all consumer food waste and cannot be readily lowered. To achieve a 50% reduction of total consumer food waste without reducing food production, ACFW would need to lower by 93%.

Ambitious policies are therefore urgently needed to put the target within reach. Because most environmental impacts of ACFW are felt domestically, most countries stand to benefit directly from national and local policy changes. However, countries bearing the largest percentage of traded environmental impacts would also see environmental benefits from halving ACFW at a global level. This can begin by developing ACFW reduction policies focused on specific commodities in accordance with national^55^ and local^56^ environmental targets that efficiently reach consumers. For instance, awareness campaigns in school canteens targeting vegetable plate waste have been effective in reducing leftovers.^57^ Focusing on rice in Southeast Asia and North Africa and on beef in the Americas could result in effective targeted measures toward reducing ACFW and its environmental impacts. Beyond specific commodities, policies could be deployed nation-wide to create food waste reduction programs in schools and workplaces, to improve expiration date labeling, and to establish mandatory food waste monitoring programs jointly with a food waste tax for food services and households. Tangible and rapid gains from these policies could then help fast-track broader policies to meet the SDG 12.3.b target. Without concrete progress in the near future, SDG 12.3.b cannot be met and the clear opportunity to reap the significant environmental benefits of reducing ACFW will be missed.

## Data Availability

All generated data are available in the main text or the Supporting Information. Secondary data used in this study are all from publicly available sources and referenced in the Materials and Methods section. Source data are provided with this paper.

## Supporting Information Available

Additional global-level results and figures, and additional methods (PDF)
Additional country-level results in table format (XLSX)
