# Supplementary material for: The Global Environmental Benefits of Halving Avoidable Consumer Food Waste
Source: Environ Sci Technol. 2024 Jul 29;58(31):13707–16. doi: 10.1021/acs.est.4c04140 (PMC11308518; doi:10.1021/acs.est.4c04140)
Supplement: Supplementary file 1 — es4c04140_si_001.pdf [file es4c04140_si_001.pdf]

The global environmental benefits of halving avoidable consumer food waste

Antoine Coudard[1,2], Zhongxiao. Sun[3*], Paul Behrens[1], José Manuel Mogollón[1]

[1] Institute of Environmental Sciences (CML), Leiden University, PO Box 9518, 2300 RA Leiden, The Netherlands
[2] Metabolic Institute, Klimopweg 150, 1032HX, Amsterdam, The Netherlands
[3] China Agriculture University, College of Land Science and Technology, 100107 Haidian District, Beijing, China

* Corresponding author:

Main contents: 1 PDF (35 pages) of Supporting Results and Methods, 26 figures (Figure S1-S26), and 17 tables (Table S17), and references

Supplementary information S1.

Supplementary Results

Harvested land impacts

Harvested land impacts: commodity contribution.

A total of 198 Mha was wasted due to avoidable consumer food waste (ACFW) in 2010. This is larger than Mexico's land surface area, the 13th largest country by land mass. Grazing for livestock represents the largest contributor (Table S1) – with ~61% of the impacts – this total can be attributed at 76% to grazing for beef cattle and sheep (incl. lamb), 13% for dairy cattle, and 9% of other meat (excl. pig and poultry).

Table S1: Top 5 land type of production wasted

Land production type	Wasted harvested areas (Mha)
Grazing	120
Wheat and products	25
Rice (Milled Equivalent)	11
Maize and products	8
Soyabeans	6

Harvested land impacts: traded impacts

Countries that waste ACFW that was produced abroad are "offshoring" the production impacts of the food abroad. Countries that produce food that then become ACFW abroad are "importing" the production impacts.

Table S2: Top 5 offshoring countries which transform imported commodities into ACFW

Country	Total 'Offshored' harvested area (Mha)
United States	4.7
China	4.0
Japan	3.9
Russia	3.5
South Korea	1.8

Table S3: Top 5 AFW commodity-country pairs with the largest offshored impacts.

Commodity	Country of waste	Country of production	'Offshored' harvested land
Cattle, Sheep, and other grazing livestock	Japan	Australia	2.4
Cattle, Sheep, and other grazing livestock	United States	Australia	2.4
Cattle, Sheep, and other grazing livestock	China	Australia	1.5
Cattle, Sheep, and other grazing livestock	Russia	Mongolia	1.4

Cattle, Sheep, and other grazing livestock	South Korea	Australia	1

Table S4: Top 5 producing countries which commodities are wasted abroad as ACFW.

**Country of production**	**'Imported' harvested areas (Mha)**	**Main wasted commodities** (>~80% of 'imported' impacts)
Australia	12.7	Cattle
United States	5.1	Cattle, Soybean, Wheat
Brazil	2.8	Cattle, Soybean
Argentina	2.3	Cattle, Soybean
Canada	2	Wheat, Cattle, Rapeseed

Harvested land impacts: domestic impacts

Table S4 and S5 presents the top countries and top commodity-country pairs for countries that both produce and waste their food domestically.

Table S4: Top 5 countries with the largest domestic impacts.

**Country of production and waste**	**Domestic harvest land (Mha)**
China	33
United States	24
Australia	12.5
Kazakhstan	10
Russia	9

Table S5: Top 5 AFW commodity-country pairs with the largest domestic impacts of food produced and wasted domestically.

**Commodity**	**Country of production and waste**	**Wasted domestic harvested land (Mha)**
Cattle, Sheep, and other grazing livestock	United States	18
Cattle, Sheep, and other grazing livestock	China	15
Cattle, Sheep, and other grazing livestock	Australia	12
Cattle, Sheep, and other grazing livestock	Kazakhstan	9
Cattle, Sheep, and other grazing livestock	China	5.5

Blue water impacts

Blue water impacts: commodity contribution.

A total of ~60 Gm^3 was wasted due to consumer avoidable food waste. This is equivalent to the water use of Brazil, the 13th largest consumer of water.

Table S6: Top 5 contributing commodities to global blue water

Commodity	Wasted blue water (Gm^3)
Rice	18.9
Wheat and products	17.2
Maize and products	3.9
Fruit, Other	2.8
Cattle	1.9

Blue water impacts: traded impacts

Table S7: Top 5 offshoring countries which transform imported commodities into ACFW

Country	Total 'Offshored' blue water (Gm^3)
United States	5.7
Japan	4.6
Russia	4.4
United Kingdom	4.2
France	3.4

Table S8: Top 5 AFW commodity-country pairs with the largest blue water offshored impacts.

Commodity	Country of waste	Country of production	'Offshored' blue water (Gm^3)
Soybean	China	United States	1.2
Rice	Saudi Arabia	Pakistan	0.9
Rice	Canada	United States	0.8
Rice	Yemen	Pakistan	0.7
Rice	Japan	United States	0.7

Table S9: Top 5 producing countries which commodities are wasted abroad as ACFW.

Country of production	Total 'Imported' blue water (Gm^3)	Main wasted commodities (>~80% of 'imported' impacts)
United States	1.9	Rice, Soybean, Maize, Wheat
Pakistan	0.9	Rice
India	0.6	Rice, Rapeseed, Wheat, Sugar cane (only > 50% of impacts)
Spain	0.5	Olive, Rice, Oranges (only > 50% of impacts)
Thailand	0.5	Rice

Blue water impacts: domestic impacts

Table S10 and S11 presents the top countries and top commodity-country pairs for countries that both produce and waste their food domestically.

Table S10: Top 5 countries with the largest domestic blue water impacts.

**Country of production and waste**	**Domestic blue water impact (Gm^3)**
China	20
India	7
United States	5.6
Egypt	2.5
Pakistan	1.9

Table S11: Top 5 AFW commodity-country pairs with the largest domestic blue water impacts of food produced and wasted domestically.

**Commodity**	**Country of production and waste**	**Domestic blue water impact (Gm^3)**
Wheat	China	8.9
Rice	China	8.8
Wheat	India	2.9
Rice	India	1.9
Fodder crops	United States	1

Greenhouse gases impacts

Greenhouse gases impacts: commodity contribution.

A total of ~396 MtCO_2eq was wasted due to consumer avoidable food waste. This is equivalent to the greenhouse gases emissions of France in 2010.

Table S12: Top 5 contributing commodities to global GHG emissions from the production of ACFW

**Commodity**	**GHG (MtCO_2eq)**
Cattle	160
Rice	82
Wheat and products	31
Pigs	20
Buffaloes	16

GHG impacts: traded impacts

Table S13: Top 5 GHG offshoring countries which transform imported commodities into ACFW

**Country**	**Total 'Offshored' GHG (MtCO_2eq)**
United States	6.4
Russia	5.4
Italy	4.7
Japan	3.6
United Kingdom	3.3

Table S14: Top 5 AFW commodity-country pairs with the largest GHG emissions offshored impacts.

**Commodity**	**Country of waste**	**Country of production**	**'Offshored' GHG emissions (MtCO_2eq)**
Cattle	Russia	Brazil	1.7
Cattle	United States	Canada	1.1
Cattle	United States	New Zealand	1
Cattle	Italy	France	0.9
Cattle	United States	Mexico	0.9

Table S15: Top 5 producing countries which commodities are wasted abroad as ACFW.

**Country of production**	**Total 'Imported' GHG emissions (MtCO_2eq)**	**Main wasted commodities** (>~50% of 'imported' impacts)
United States	7.7	Cattle, Rice, Wheat
Brazil	7.0	Cattle
India	6.6	Cattle, Buffaloes
Australia	4.3	Cattle, Sheep
France	3.4	Cattle, Wheat

GHG impacts: domestic impacts

Table S16 and S17 presents the top countries and top commodity-country pairs for countries that both produce and waste their food domestically.

Table S16: Top 5 countries with the largest domestic blue water impacts.

Country of production and waste	Domestic GHG impact ($MtCO_2eq$)
China	95
United States	34
Brazil	24
India	23
Russia	12

Table S17: Top 5 AFW commodity-country pairs with the largest domestic GHG impacts of food produced and wasted domestically.

Commodity	Country of production and waste	Domestic GHG impact ($MtCO_2eq$)
Rice	China	42
Cattle	Brazil	21
Cattle	United States	19
Cattle	China	11
Vegetables, Other	China	7.3

Supplementary Figures

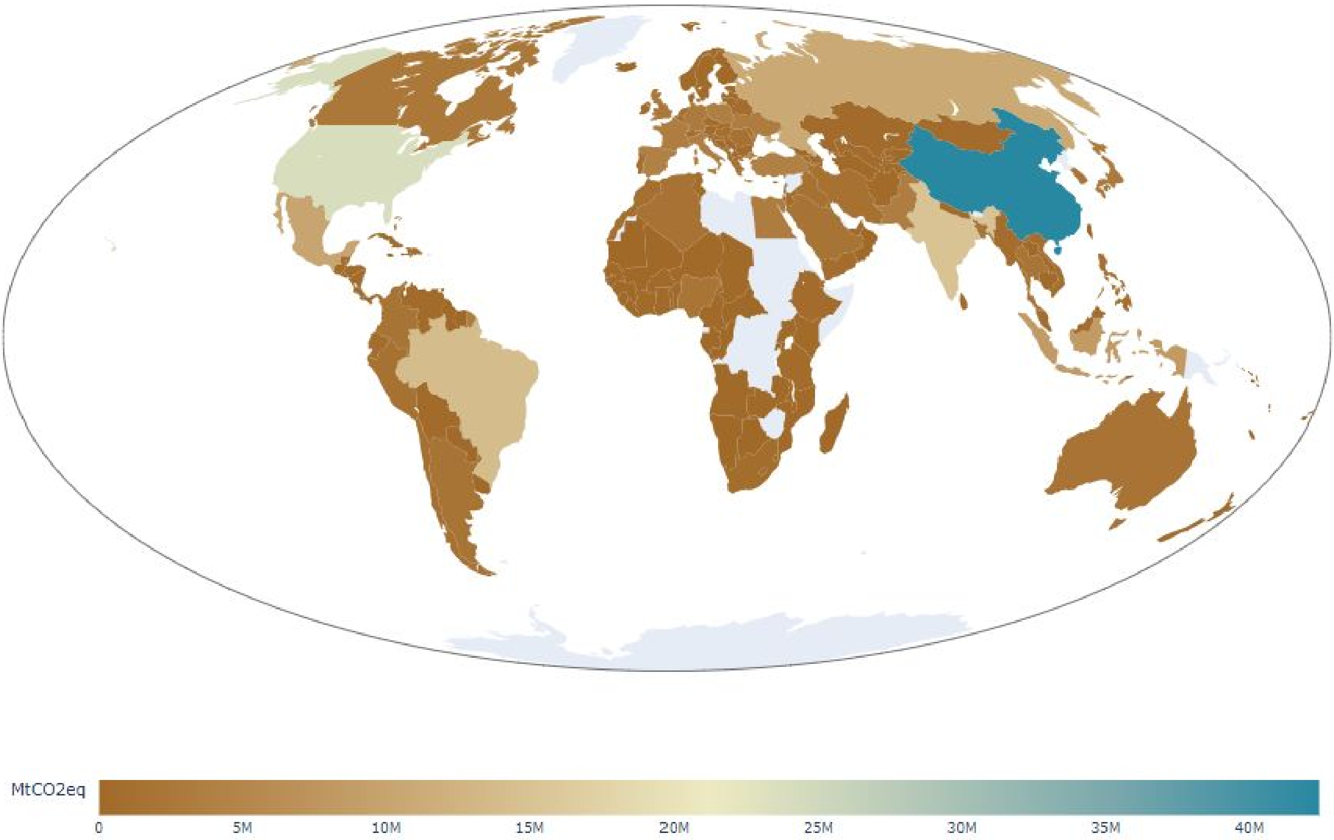

Figure S1: GHG emissions avoided by avoiding the waste treatment of half of avoidable consumer food waste. – in tonnes CO2eq.

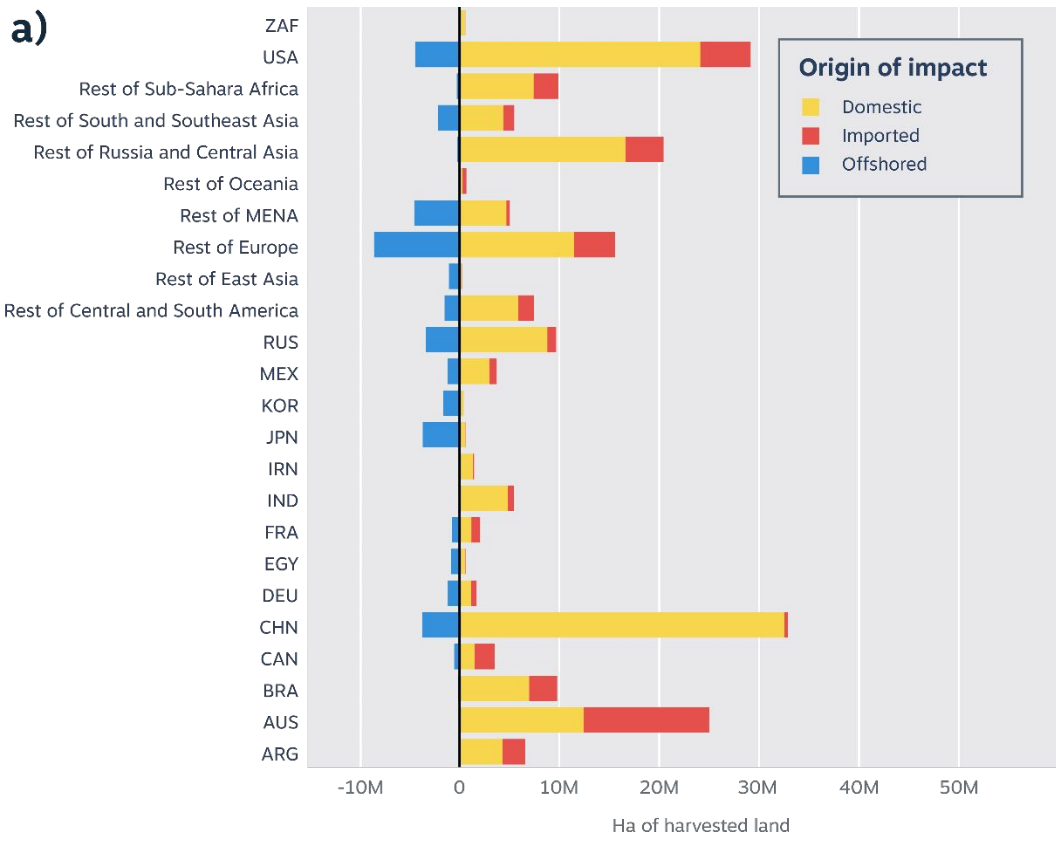
a)
ZAF
USA
Rest of Sub-Sahara Africa
Rest of South and Southeast Asia
Rest of Russia and Central Asia
Rest of Oceania
Rest of MENA
Rest of Europe
Rest of East Asia
Rest of Central and South America
RUS
MEX
KOR
JPN
IRN
IND
FRA
EGY
DEU
CHN
CAN
BRA
AUS
ARG
-10M
0
10M
20M
30M
40M
50M
Ha of harvested land
Origin of impact
Domestic
Imported
Offshored

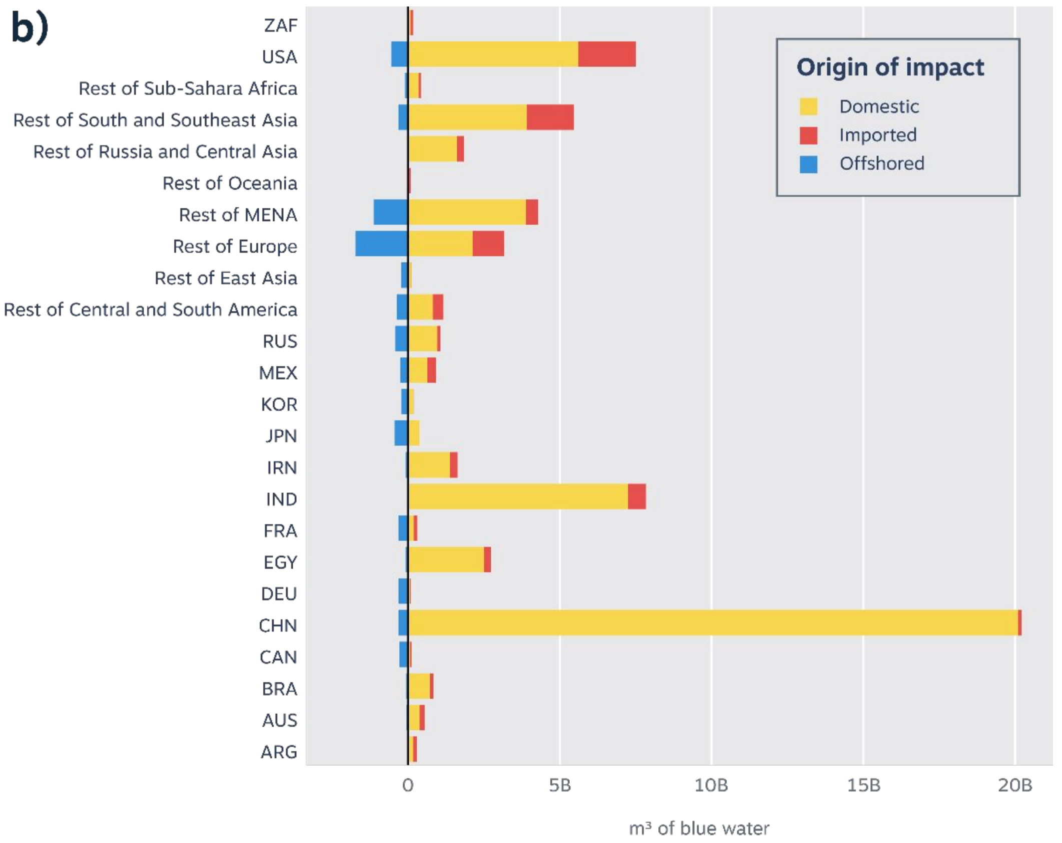
b)
ZAF
USA
Rest of Sub-Sahara Africa
Rest of South and Southeast Asia
Rest of Russia and Central Asia
Rest of Oceania
Rest of MENA
Rest of Europe
Rest of East Asia
Rest of Central and South America
RUS
MEX
KOR
JPN
IRN
IND
FRA
EGY
DEU
CHN
CAN
BRA
AUS
ARG
0
5B
10B
15B
20B
m³ of blue water
Origin of impact
Domestic
Imported
Offshored

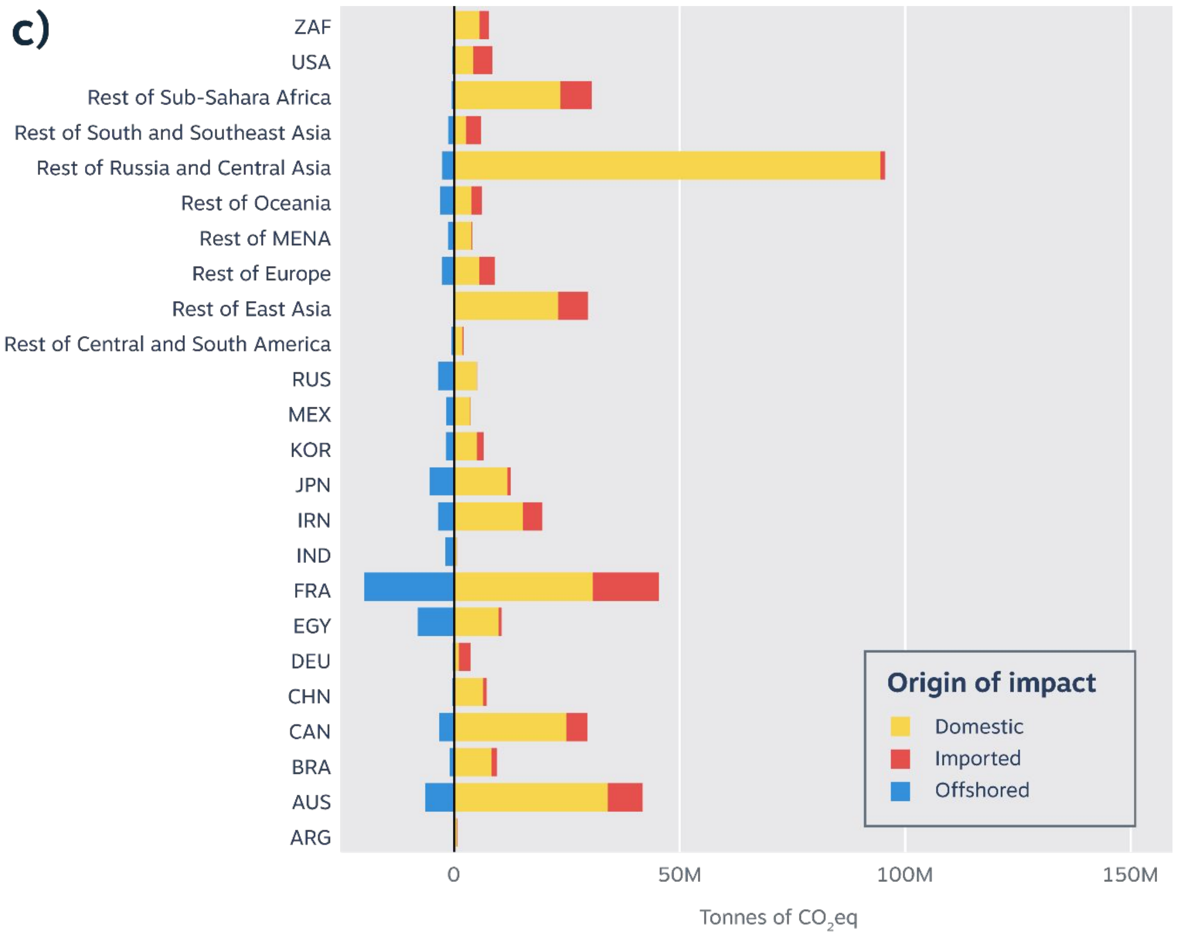

Figure S2: a) ACFW harvested land impact origin. b) ACFW blue water impact origin. c) ACFW GHG emissions impact origin. These figures are the same as in main manuscript Fig1 a,b,c – in larger size.

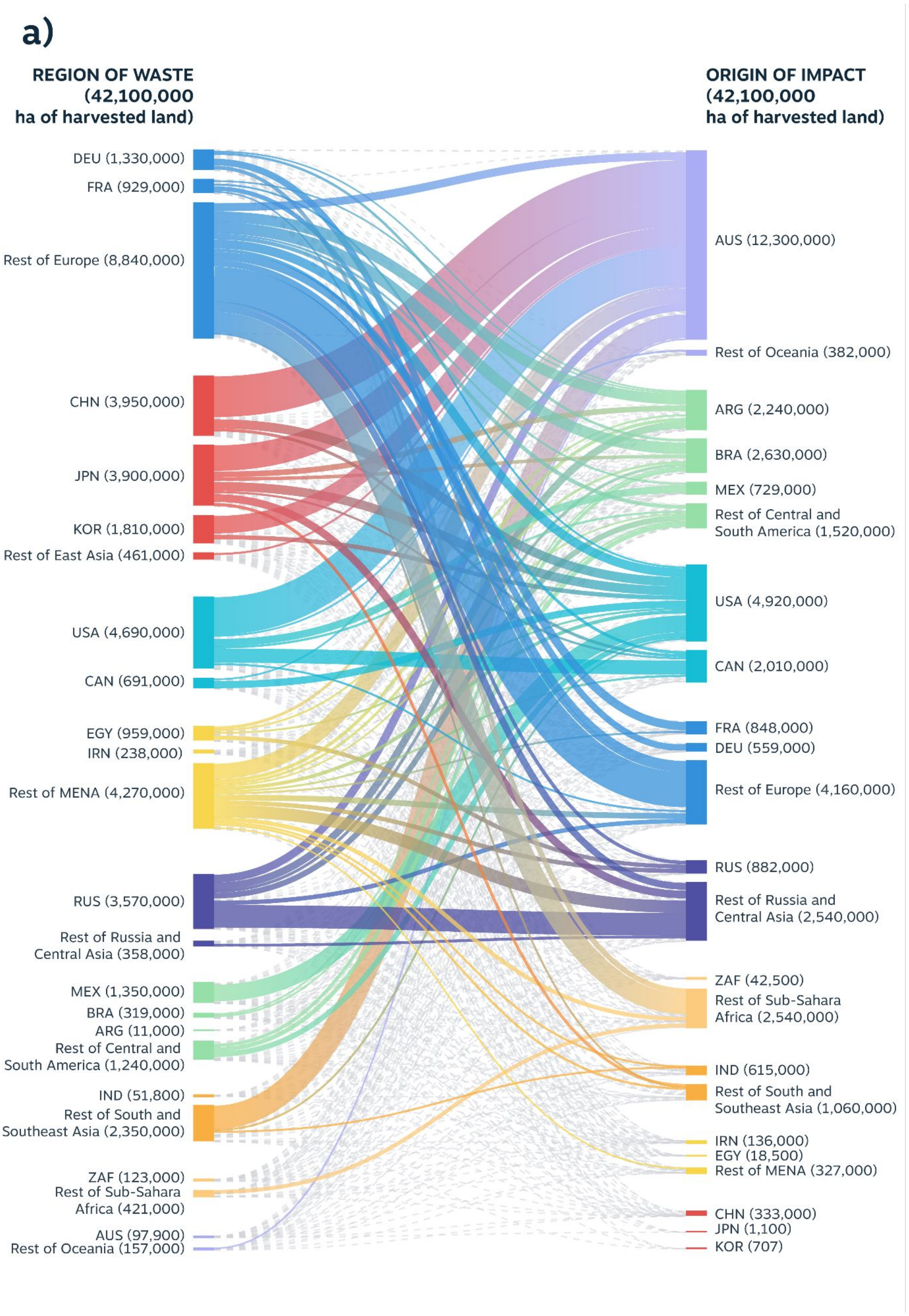
a)
REGION OF WASTE
(42,100,000
ha of harvested land)
ORIGIN OF IMPACT
(42,100,000
ha of harvested land)
DEU (1,330,000)
FRA (929,000)
Rest of Europe (8,840,000)
CHN (3,950,000)
JPN (3,900,000)
KOR (1,810,000)
Rest of East Asia (461,000)
USA (4,690,000)
CAN (691,000)
EGY (959,000)
IRN (238,000)
Rest of MENA (4,270,000)
RUS (3,570,000)
Rest of Russia and
Central Asia (358,000)
MEX (1,350,000)
BRA (319,000)
ARG (11,000)
Rest of Central and
South America (1,240,000)
IND (51,800)
Rest of South and
Southeast Asia (2,350,000)
ZAF (123,000)
Rest of Sub-Sahara
Africa (421,000)
AUS (97,900)
Rest of Oceania (157,000)
AUS (12,300,000)
Rest of Oceania (382,000)
ARG (2,240,000)
BRA (2,630,000)
MEX (729,000)
Rest of Central and
South America (1,520,000)
USA (4,920,000)
CAN (2,010,000)
FRA (848,000)
DEU (559,000)
Rest of Europe (4,160,000)
RUS (882,000)
Rest of Russia and
Central Asia (2,540,000)
ZAF (42,500)
Rest of Sub-Sahara
Africa (2,540,000)
IND (615,000)
Rest of South and
Southeast Asia (1,060,000)
IRN (136,000)
EGY (18,500)
Rest of MENA (327,000)
CHN (333,000)
JPN (1,100)
KOR (707)

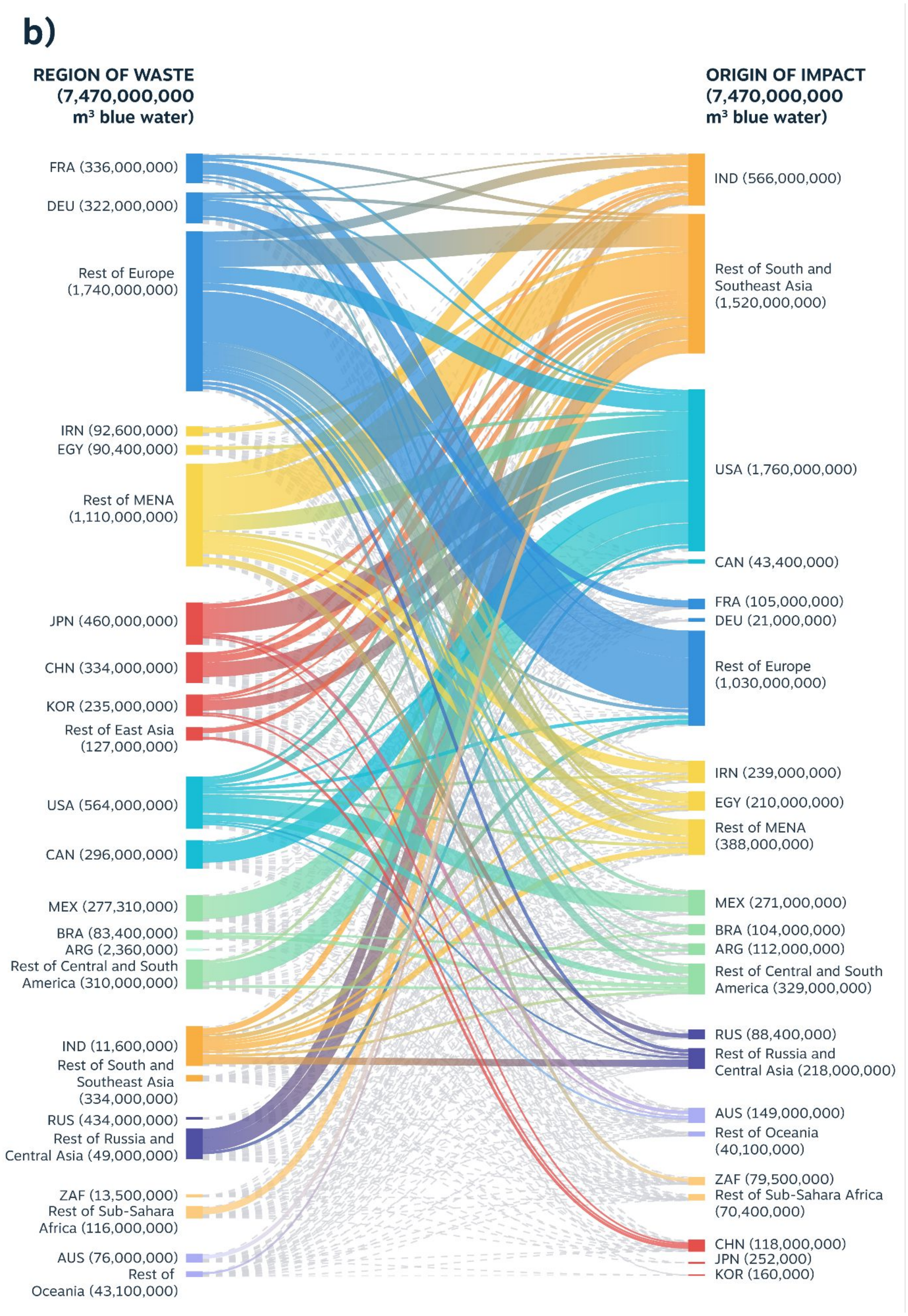
b)
REGION OF WASTE (7,470,000,000 m³ blue water)
ORIGIN OF IMPACT (7,470,000,000 m³ blue water)
FRA (336,000,000)
DEU (322,000,000)
Rest of Europe (1,740,000,000)
IRN (92,600,000)
EGY (90,400,000)
Rest of MENA (1,110,000,000)
JPN (460,000,000)
CHN (334,000,000)
KOR (235,000,000)
Rest of East Asia (127,000,000)
USA (564,000,000)
CAN (296,000,000)
MEX (277,310,000)
BRA (83,400,000)
ARG (2,360,000)
Rest of Central and South America (310,000,000)
IND (11,600,000)
Rest of South and Southeast Asia (334,000,000)
RUS (434,000,000)
Rest of Russia and Central Asia (49,000,000)
ZAF (13,500,000)
Rest of Sub-Sahara Africa (116,000,000)
AUS (76,000,000)
Rest of Oceania (43,100,000)
IND (566,000,000)
Rest of South and Southeast Asia (1,520,000,000)
USA (1,760,000,000)
CAN (43,400,000)
FRA (105,000,000)
DEU (21,000,000)
Rest of Europe (1,030,000,000)
IRN (239,000,000)
EGY (210,000,000)
Rest of MENA (388,000,000)
MEX (271,000,000)
BRA (104,000,000)
ARG (112,000,000)
Rest of Central and South America (329,000,000)
RUS (88,400,000)
Rest of Russia and Central Asia (218,000,000)
AUS (149,000,000)
Rest of Oceania (40,100,000)
ZAF (79,500,000)
Rest of Sub-Sahara Africa (70,400,000)
CHN (118,000,000)
JPN (252,000)
KOR (160,000)

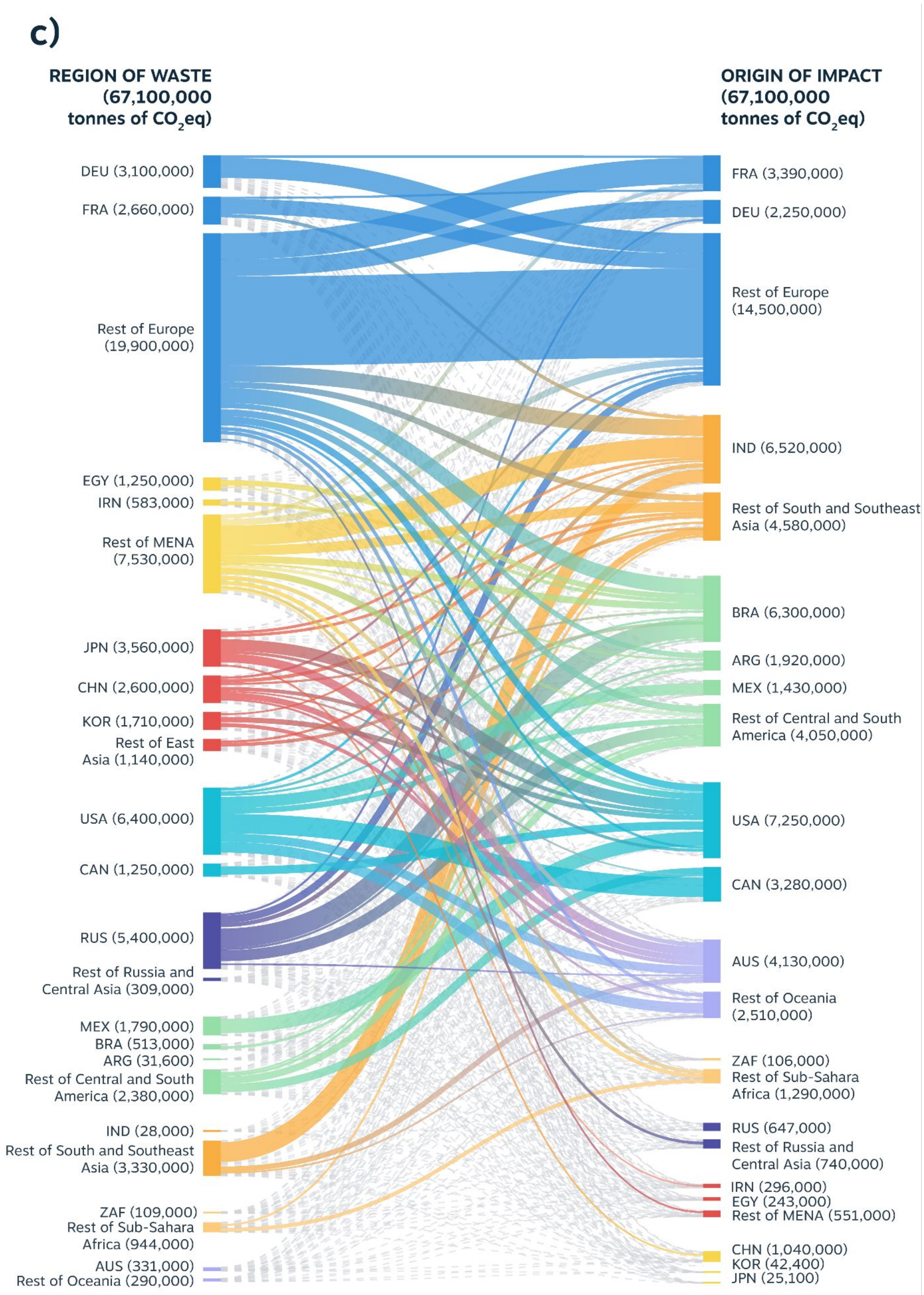

Figure S3: a) Traded harvested land. b) Traded blue water. c) Traded GHG - of ACFW. These figures are the same as in main manuscript Fig2 a,b,c – in larger size.

Country-specific Figures

Harvested land

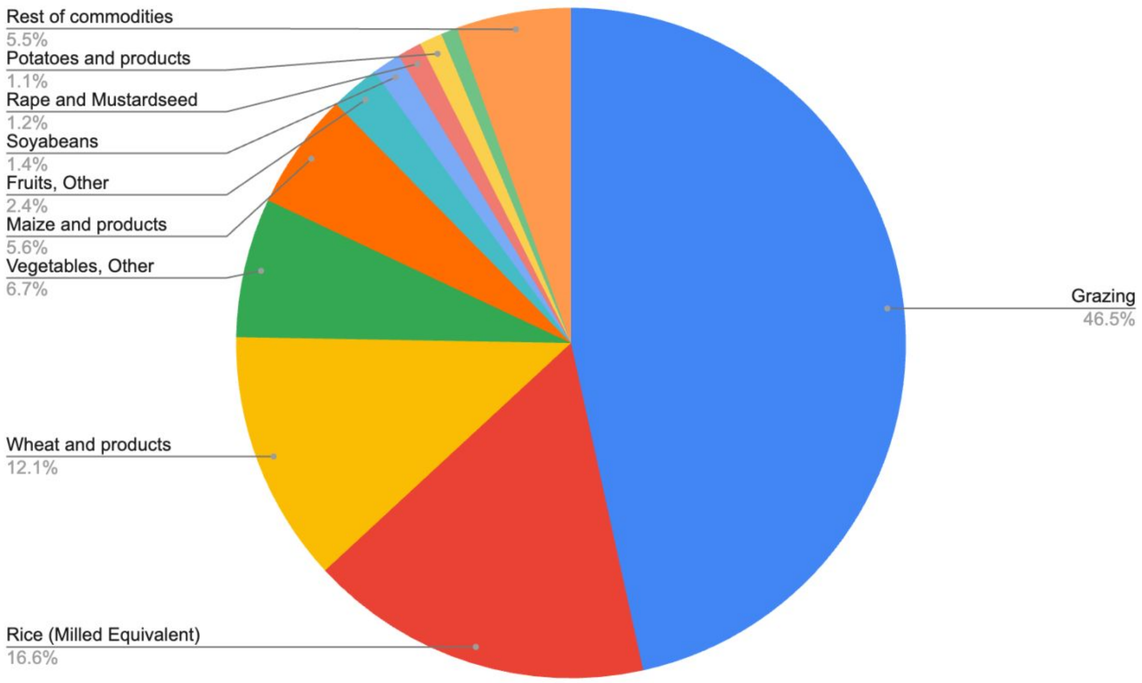

Figure S4 – Commodity contribution to domestic ACFW land impacts in China

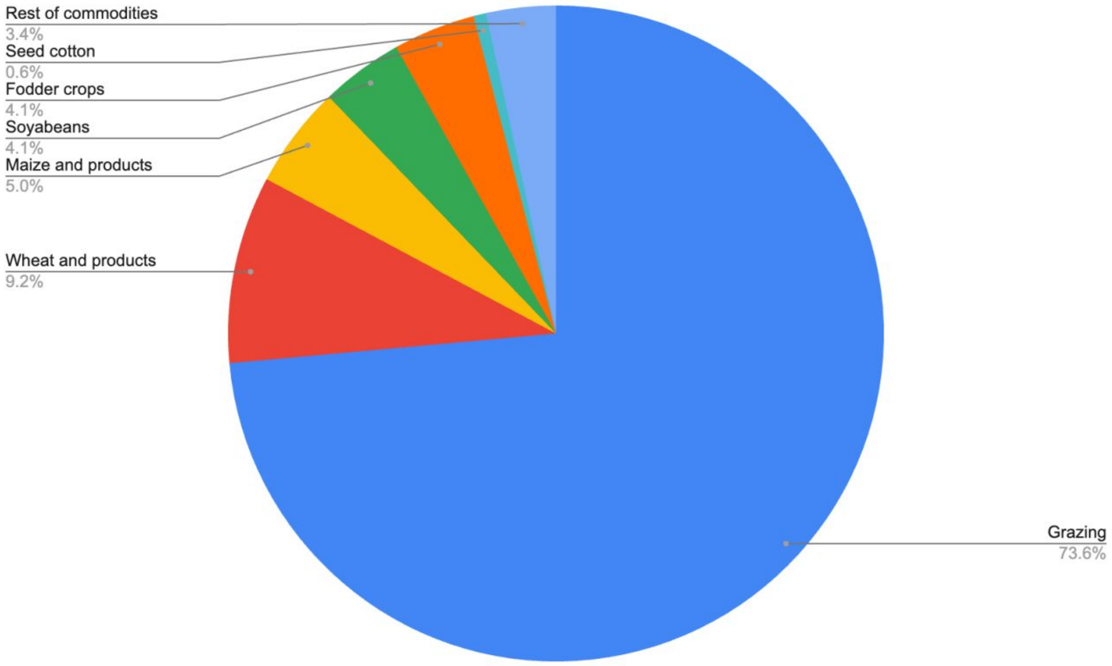

Figure S5 – Commodity contribution to domestic ACFW's land impacts in the USA

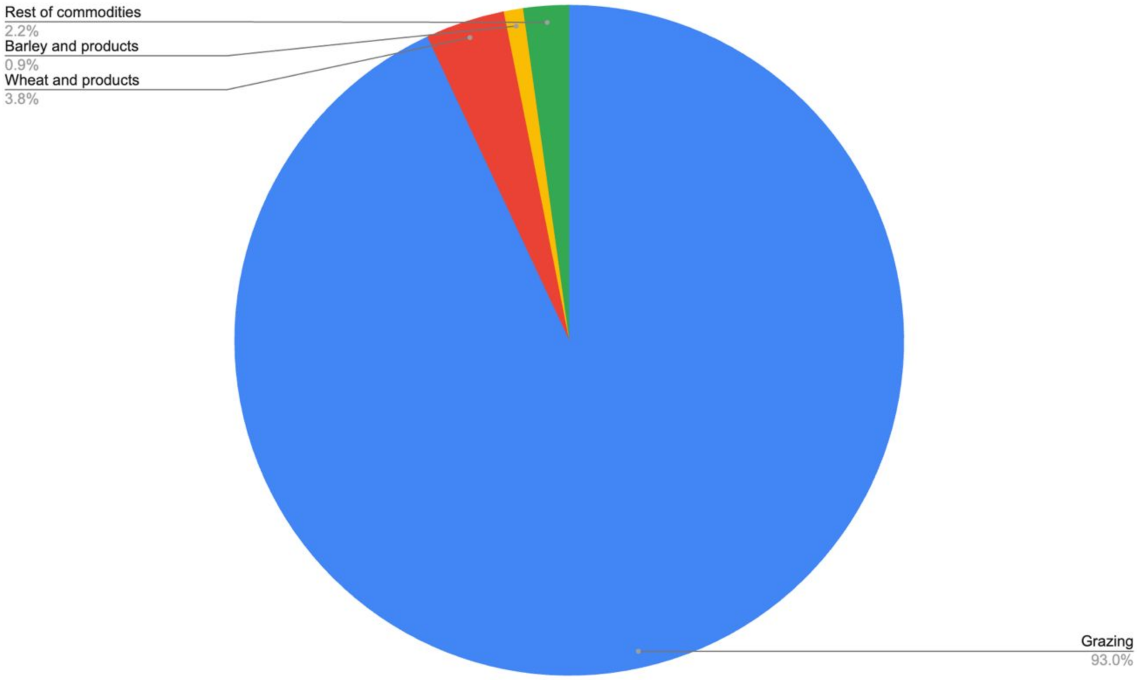

Figure S6 – Commodity contribution to domestic ACFW’s land impacts in Australia

GHG emissions

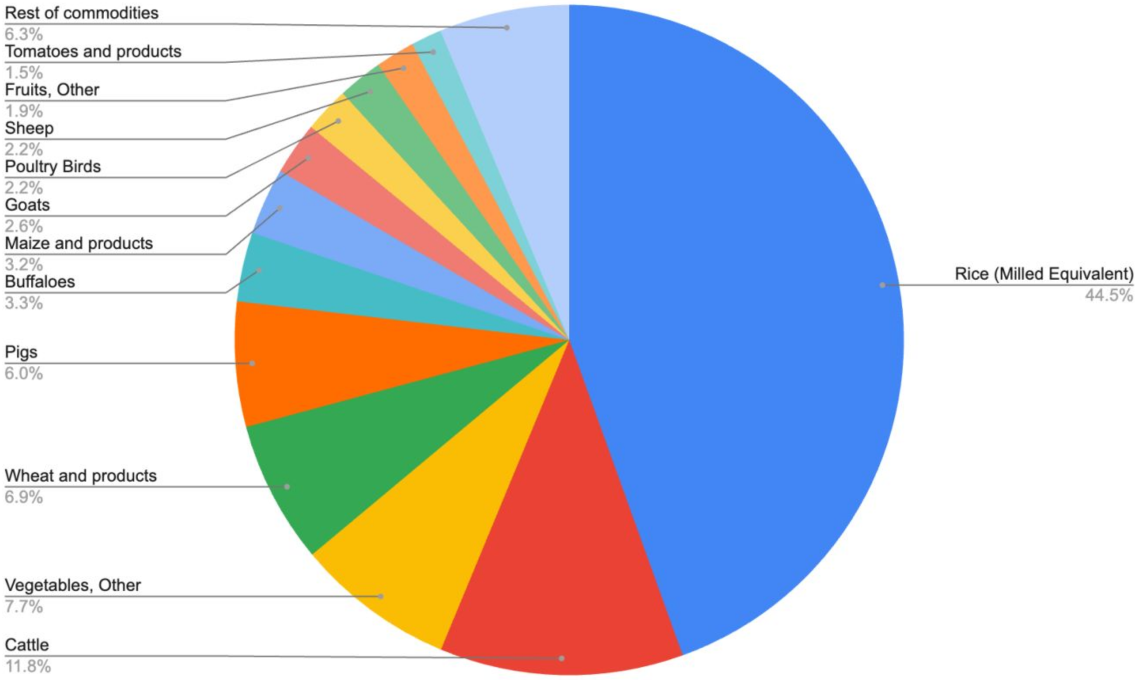

Figure S7 – Commodity contribution to domestic ACFW’s GHG impacts in China

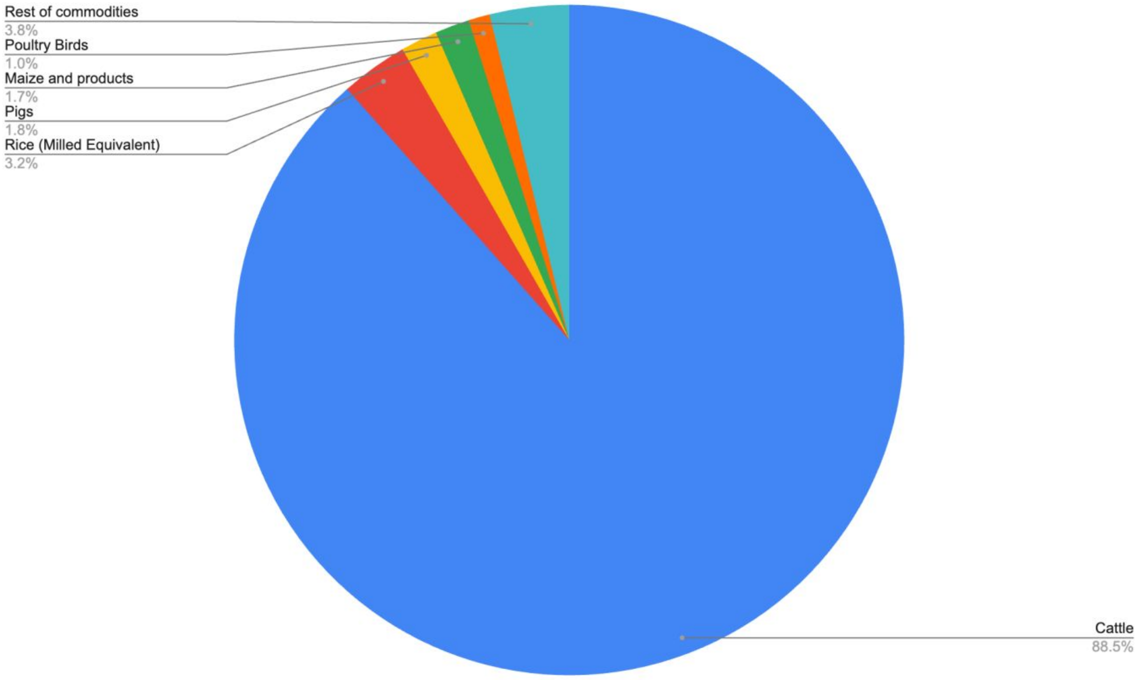

Figure S8 – Commodity contribution to domestic ACFW's GHG impacts in Brazil

Blue Water

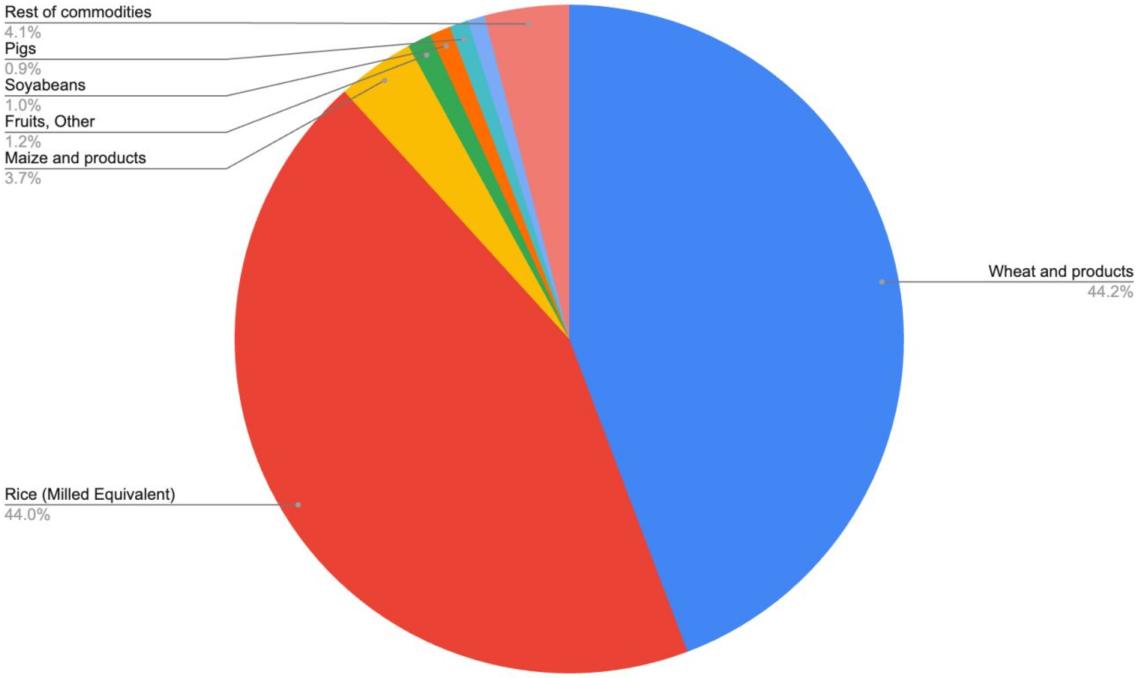

Figure S9 – Commodity contribution to domestic ACFW's blue impacts in China

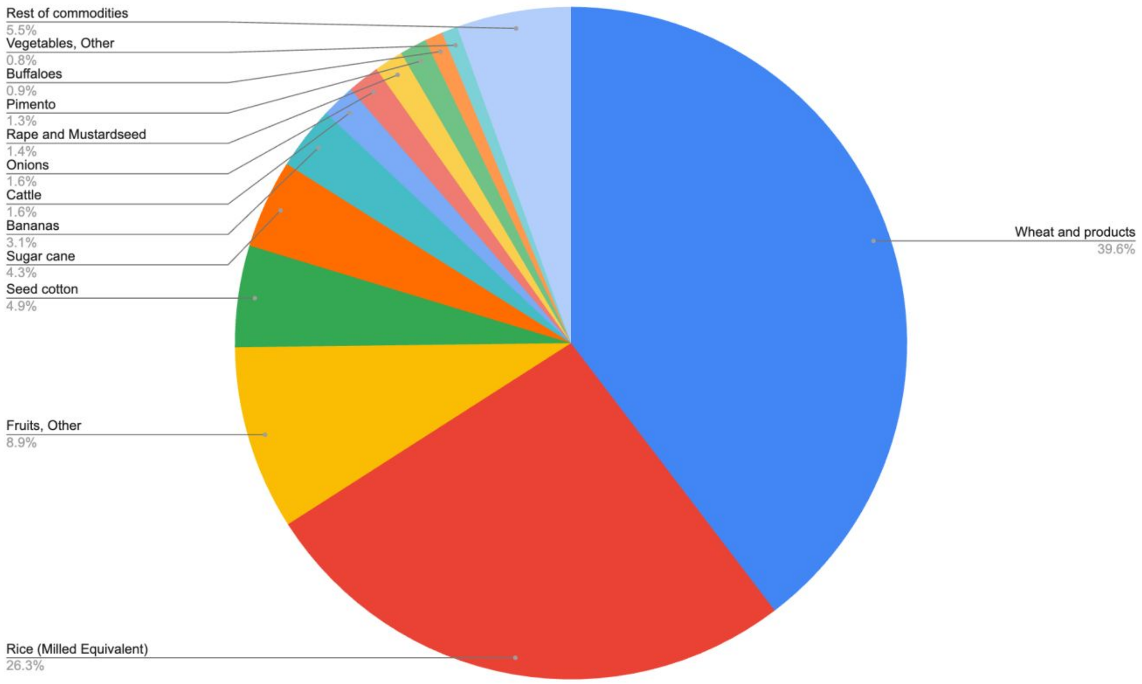

Figure S10 – Commodity contribution to domestic ACFW's blue impacts in India

Supplementary Methods

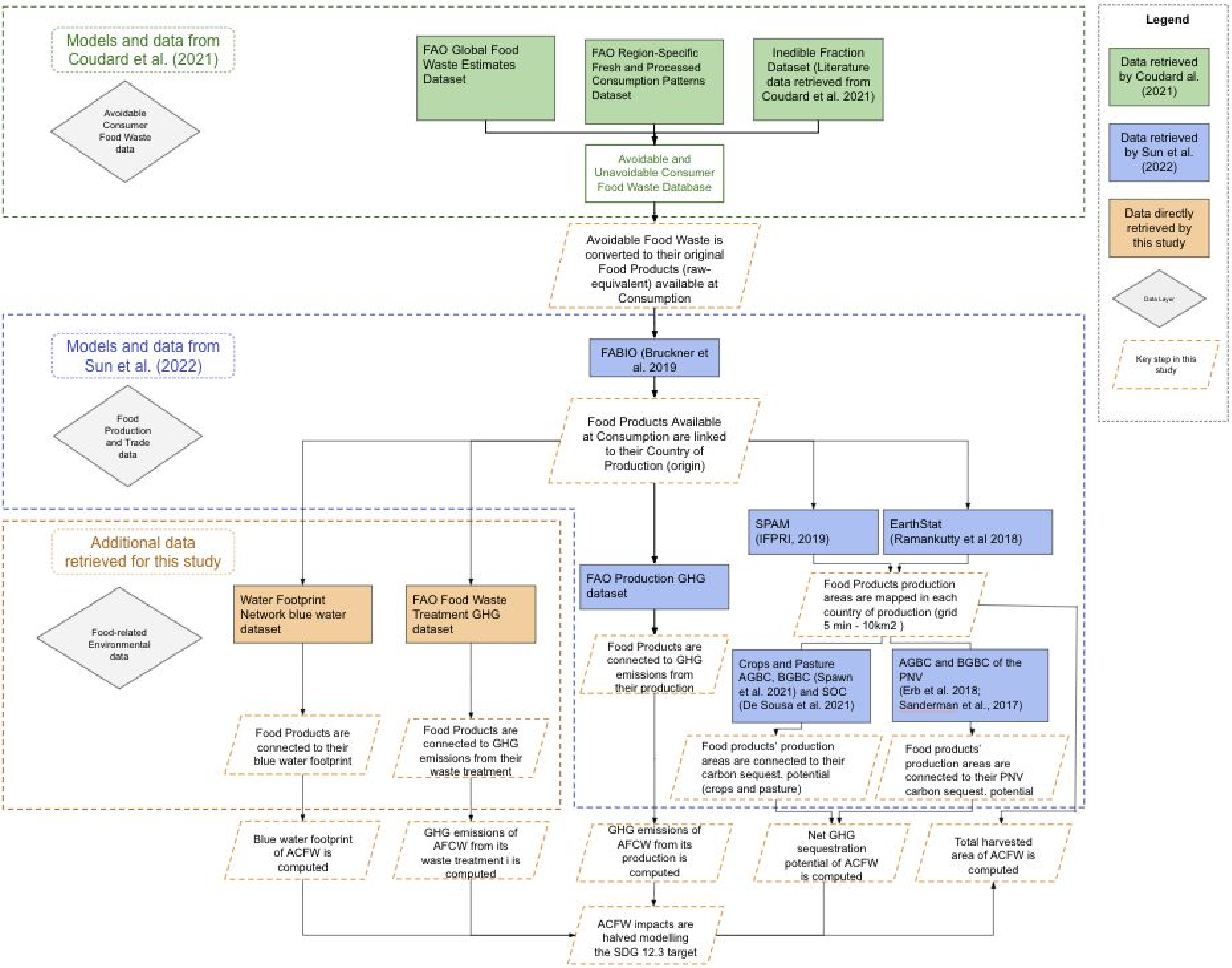

Fig. S11. Workflow of this Study

Avoidable Consumer Food Waste Model

Quantifying Food Availability at the Household and Food Services Level

The overall workflow of the model development was developed in Coudard et al. (2021)[1]. It starts with the download of FAO Food Balance Sheets for each country and ends with a model quantifying Avoidable Food Waste (AFW) at Consumption stage for each country. Further details and discussion on the model are provided in Coudard et al.[1]

The initial step involves quantifying the quantities of accessible food during the consumption phase, which includes households and food services. This model utilizes FAOSTAT[2] Food Balance Sheets (FBSs), which compile the available food at the distribution phase within a given country. This compilation accounts for losses occurring earlier in the supply chain, as well as the utilization of food products for purposes other than human consumption (such as

seeds, feed, etc.), and exports. Consequently, the FBSs offer an average measure of food supply at the national level for each country. This measurement is presented in kilograms per capita per annum, encompassing around 90 subtypes of food products or 18 aggregated categories of food[2]. Notably, FAOSTAT reports the availability of food in primary-equivalent terms. Consequently, for processed items, the compiled quantities are expressed in primary-equivalent, which might appear greater than the practical amounts found at the retail level. For instance, products like pasta or bread are quantified as their wheat-equivalent[3].To accurately assess the actual quantities of available food, the FBSs need to be adjusted using technical conversion factors (TCFs) in line with the methodology outlined by Vanham et al[4]. This adjustment is crucial for food waste calculations. In this study, we employ the TCFs initially developed by FAO[4] and compiled by Bruckner et al[5]. The TCFs supplied by FAO are tailored to the most finely detailed food items recorded in their data. The TCFs are therefore used to calculate the product-equivalent from the primary-equivalent data of the FBSs (Eq. 1).

$$\boldsymbol{FA}_{\ f} = \boldsymbol{FA}_{f\,PE} * \boldsymbol{TCF}_f$$

Eq. 1

Where:
$\boldsymbol{FA}_f$ is the corrected, actual quantity of a food item f available at the Distribution stage, in kg
$\boldsymbol{FA}_{\boldsymbol{f}\,\boldsymbol{PE}}$ is the primary-equivalent quantity of food item f, compiled in the FBS, in kg
$\boldsymbol{TCF}_{\boldsymbol{f}}$ is the Technical Conversion Factor of food item f, as a percentage.

This step yields the actual amount of food available at the retail-level. Prior to reaching their ultimate consumption point, which includes both food services and households, food products might also be wasted during the Distribution stage. This wastage essentially reduces the quantity of food that effectively makes it to the Consumption stage. Consequently, the subsequent step involves accounting for these losses that occur before consumption.

To calculate the losses during the Distribution stage, it's important to consider the nature of the food products—whether they are processed or fresh—since they exhibit varying rates of food waste incidence[6]. Consequently, FAO provides distinct estimates for food waste, depending on whether a food type is consumed in a processed or fresh state. This differentiation concerning the nature of products applies to the following grouped food categories: Vegetables, Fruits, Starchy Roots, and Fish and Seafood, with estimates available for various regions across the globe. These regional waste estimates are detailed in the comprehensive FAO Global Food Estimates report. This report presents the incidence of food waste for different world regions during the Distribution stage (retail level) for diverse food groups, considering whether they are processed or fresh. Aligning the more aggregated food categories used in the Global Food Estimates with the 18 consolidated food groups in the FBSs requires a harmonization of food item classifications. This harmonization is applied to the 90 disaggregated food items.

The actual quantities of food that ultimately reach households and food services in each country are then calculated. This involves first determining the overall amount of both processed and fresh food that is wasted during the Distribution stage (using Equations 2 and 2.bis). These calculated losses are then subtracted from the total to obtain the actual quantities of food that consumers ultimately receive (as described in Equation 3). The losses are quantified for both Fresh and Processed products for the relevant food types.

$$\boldsymbol{FWD}_{f\,FRESH} = \boldsymbol{FA}_f * \boldsymbol{CT\ share}_{\boldsymbol{FRESH}} * \boldsymbol{DWF}_{F\,FRESH}$$

Eq. 2

Where:

- $\boldsymbol{FWD}_{f\ FRESH}$ is the quantity of a food item f, consumed fresh, wasted at the Distribution stage, in kg.
- $\boldsymbol{CT\ share}_{\boldsymbol{FRESH}}$is the consumption type share of food item f considered to be consumed fresh (without any processing), as a percentage.
- $\boldsymbol{DWF}_{F\ FRESH}$ is the Distribution food waste factor from $Food\ Group\ F$ to which food item f belong and applied to food item f.

$$\boldsymbol{FWD}_{f\ PROCESSED} = \boldsymbol{FA}_{f} * \boldsymbol{CT\ share}_{PROCESSED} * \boldsymbol{DWF}_{F\ PROCESSED}$$

Eq. 2.bis

Where:

- $\boldsymbol{FWD}_{f\ PROCESSED}$ is the quantity of a food item f, consumed fresh, wasted at the Distribution stage, in kg
- $\boldsymbol{CT\ share}_{PROCESSED}$is the consumption type share of food item f considered to be consumed processed, as a percentage.
- $\boldsymbol{DWF}_{F\ PROCESSED}$ is the Distribution food waste factor from $Food\ Group\ F$, considered to be processed, to which food item f belong and applied to food item f.

The amounts of food available to consumers can then be derived with the following equations.

$$\boldsymbol{FAC}_{f\ FRESH} = \mathbf{FA}_{f} * \boldsymbol{CT\ share}_{\boldsymbol{FRESH}} - \boldsymbol{FWD}_{f\ FRESH}$$

$$\boldsymbol{FAC}_{f\ \ PROCESSED} = \mathbf{FA}_{f} * \boldsymbol{CT\ share}_{\boldsymbol{FRESH}} - \boldsymbol{FWD}_{f\ PROCESSED}$$

Eq. 3 and 3 bis

Where:

- $\boldsymbol{FAC}_{f\ FRESH}$ is the quantity of a food item f, fresh, available at the Consumption stage (food services and households), in kg
- $\boldsymbol{FA}_{f}$ is the corrected, actual quantity of a food item f available at the Distribution stage, in kg
- $\boldsymbol{CT\ share}$ is the consumption type share of food item f considered to be consumed, whether fresh or processed, as a percentage.
- $\boldsymbol{FWD}_{f}$ is the quantity of a food item f, consumed fresh or processed, wasted at the Distribution stage, in kg.

Quantifying Avoidable Consumer Food Waste

Avoidable Consumer Food Waste (ACFW) is calculated by combining FAO *Global Food Estimate*s at the consumption stage for every country, with their respective regional estimates. The food waste estimates take into consideration both ACFW and Unavoidable Consumer Food Waste (UCFW) (e.g., sheel, peels, seed). To accurately calculate ACFW, the UCFW fraction must be subtracted from the total calculated food waste at consumption stage. Quantifying the total UCFW is achieved through the "waste floor" approach. The "waste floor"

approach aims to quantify the minimum volume of UCFW generated by each country. It calculates the total "minimal" volume of UCFW associated with the final consumption of food within households and food services[7]. To accomplish this, we employ data from De Laurentiis et al.[7] for vegetables, fruits, and starchy roots. Additionally, WRAP[8] provides estimates for the meat group and its subcategories (bovine, pork, poultry, sheep), as well as fish and seafood. Furthermore, it's assumed that stimulants like coffee and tea grounds constitute 100% of UCFW. Inedible fraction estimates for eggshells[9] are also incorporated. A core assumption in this approach is to consider that processed food products (such as cans, jars, frozen, juice, dried) will be entirely edible, and therefore will not generate any UCFW (Eq. 4bis), as they had the inedible portions removed at the processing stage. It is also coherent with the "waste floor" approach seeking to quantify the *minimal* amounts of UFW. As a result, the inedible fractions of the relevant food products are matched with their respective food groups. The cumulative volume of UFW for each country is then quantified by multiplying the fraction with the total available quantities of (fresh) food products post-distribution (as described in Eq.4).

$$\boldsymbol{UCFW}_{f\ FRESH} = \boldsymbol{FAC}_{f\ FRESH} * \boldsymbol{IF}_{f\ FRESH}$$

Eq. 4

Where:
- $\boldsymbol{UCFW}_f$ is the unavoidable food waste quantity of a food item f, consumed fresh, that is generated at the Consumption stage, in kg
- $\boldsymbol{FAC}_{f\ FRESH}$ is the quantity of a food item f, fresh, available at the Consumption stage (food services and households), in kg
- $\boldsymbol{IF}_{f\ FRESH}$is the inedible fraction of food item f, consumed fresh, as a percentage.

$$\boldsymbol{UFW}_{f\ PROCESSED} = \mathbf{0}$$

Eq. 4.bis

Where:
- $\boldsymbol{UFW}_{f\ PROCESSED}$ is considered to be 0 as the processed food item f is considered to have been stripped of the inedible, or unavoidable waste elements.

This step results in the amounts of UCFW for each country.

To obtain the final amounts of ACFW, the inedible shares of food products must also be considered, and subtracted from the total amounts of food waste (ACFW+UCFW) that are themselves calculated via the FAO Global Food Estimates (Eq. 5).

$$\boldsymbol{FWC}_{f\ FRESH} = \boldsymbol{FAC}_{f\ FRESH} * \boldsymbol{CWF}_{F\ FRESH}$$

Eq. 5

Where:
- $\boldsymbol{FWC}_{f\ FRESH}$ is the quantity of a food item f, consumed fresh, wasted at the Consumption stage, in kg.
- $\boldsymbol{FAC}_{f\ FRESH}$ is the quantity of a food item f, fresh, available at the Consumption stage (food services and households), in kg
- $\boldsymbol{CWF}_{F\ FRESH}$ is the Consumption food waste factor from $Food\ Group\ F$ to which food item f belong, and applied to food item f.

$$ACFW_{f\ FRESH} = FWC_{f\ FRESH} * (1 - IF_{f\ FRESH})$$

Eq. 6

Where:

- $ACFW_{f\ FRESH}$ is the edible quantity of a food item f, consumed fresh, that is wasted at the Consumption stage, in kg
- $FWC_{f\ FRESH}$ is the quantity of a food item f, consumed fresh, wasted at the Consumption stage, in kg.
- $IF_{f\ FRESH}$is the inedible fraction of food item f, consumed fresh, as a percentage.

For processed food item f, as $IF_{f\ PROCESSED} = 0$, the following relationship can be derived:

$$ACFW_{f\ PROCESSED} = FWC_{f\ PROCESSED}$$

Eq. 7

This step yields the total amounts of ACFW for each food type for every country compiled in the FBSs. It should be noted that some limitations exist around the food waste factors used in this model. Food waste estimates are inherently uncertain due to the difficulty of data collection and the geographic and temporal coverages of datasets[10]. Nonetheless, the FAO waste factors have been developed based on the best available knowledge. Uncertainties around food waste estimate are further explored in Coudard et al.[1].

Harvested land, GHG emissions, and blue water from production of ACFW

We integrated the ACFW model with spatially explicit multi-regional input-output model from Sun et al.[11] to assess harvested land, GHG emissions, and blue water consumption of the different food commodities. The MRIO model uses the Food and Agriculture Biomass Input–Output dataset (FABIO) from Bruckner et al.[6] in physical units that relates the international final demand for food items with primary agricultural. The model select data for the year 2010 from FABIO.

FABIO covers 191 countries and 128 agricultural, food, and forestry products from 1986 to 2013. Data on food items are then combined and harmonized with the global dataset on consumer avoidable food waste from Coudard et al. that reports total quantities of consumer avoidable food waste for 192 countries. Since it is also based on the same nomenclature of FAOSTAT, the avoidable food waste items were readily matched to FABIO food items. As a result, avoidable food waste at the consumer level can be related back to the countries of their primary agricultural production. Bruckner et al.[6] provide further details on the construction of FABIO.

In the FABIO model, Brucker et al. acknowledge the potential issue of re-exports within IO and trade data. However, they propose a methodology to mitigate this concern. The model assumes that importers are more likely to accurately report the origin of traded commodities, as opposed to exporters determining the final destination. Import statistics are often more comprehensive due to customs' vested interest in accurate data collection for taxation purposes. To address missing records, the FABIO model utilizes 'mirror' statistics from trade partners. This approach supplements incomplete data sets, ensuring a more comprehensive representation of trade flows. To verify that trade data reflects the final country of consumption,

the FABIO model employs detailed trade statistics that track the origin and destination of goods. It distinguishes between intermediate and final goods, aligning trade flows with actual consumption locations. By tracing the movement of goods through international supply chains, from production to end-use, the FABIO model provides a precise of consumption-based demand for resources across different countries.

The harvested area used to grow the avoidable food waste is quantified using FAOSTAT crop and pasture area data, combined with SPAM, a spatial production allocation model[12] for 29 herbaceous crops and EarthStat[13], a spatially explicit cropland and pastureland information dataset for the fodder crops. This step enables us to quantify the spatially explicit environmental impacts from the production of food commodities that will become ACFW. A GHG emissions dataset derived from FAOSTAT[4] at the national level is also linked to FABIO to quantify emissions from the agricultural activities that occur to produce the food items that ultimately become consumer avoidable food waste. The GHG emissions estimates, retrieved from Sun et al., were built using an older version of 100-year Global Warming Potentials (GWP), with those from the IPCC Fifth 5 Assessment Report (AR5)[14] with climate-carbon feedback (that is, 34 CO2e for CH4 and 298 66 CO2e for N2O). The same process is performed to quantify the blue water use during the agricultural production stage of the commodities, using datasets from the Water Footprint Network[15] for crop products and FAOSTAT for livestock products[16]. The calculated impacts of the ACFW are allocated to each production country based on the share of the sourcing country-mix of each consuming (and wasting) country. As such, if Country A sources 25% of its final consumption of beef products from Country B (the original producer), as a result 25% of the wasted harvested land used to produce the beef wasted in Country A is allocated to Country B.

Halving avoidable consumer food waste

We use the UN SDG 12.3.b target as a basis for to model a reduction in avoidable food waste and to estimate the amounts of land that could potentially be restored to their PNV. The simplified approach halves ACFW (50% reduction) across all food categories in every country. The avoidable food waste reduction scenario is of course highly idealized – as it is meant to explore the potential magnitude of such a shift on global natural resources and GHG emissions. The total environmental impacts (land use, blue water, and GHG emissions) that occurred during the production of ACFW are therefore halved.

GHG emissions reduction from the avoided ACFW End-of-Life

A GHG emissions dataset derived from FAOSTAT links the total quantities of food waste generated in each country with the total GHG emissions from the waste treatment activities (e.g., landfill), in tCO_2eq. This dataset was developed by Crippa et al.[17]. This model determined the waste treatment activities based on data from the *WhataWaste2.0* dataset developed by the World Bank[18]. This dataset covers municipal waste from retail, food services and consumers, and therefore excludes industrial waste from agriculture and food processing. For each country, the total food waste reaching the municipal waste treatment activities is there defined as:

$$FW_{municipal\ waste\ treatment} = RFW + ACFW + UCFW$$

Eq. 8

Where:

- $FW_{municipal\ waste\ treatment}$ is the total amount of food waste reaching municipal waste treatment activities in the given country.
- RFW is the quantity of a food waste from food retail, reaching municipal waste treatment activities.
- $ACFW$ is the quantity of a avoidable consumer food waste reaching municipal waste treatment activities.
- $UCFW$ is the quantity of a unavoidable consumer food waste reaching municipal waste treatment activities.

We then isolate the mass share of ACFW relative the total food waste reaching municipal waste treatment, for each country.

$$ACFW_{share} = AFWC/FW_{municipal\ waste\ treatment}$$

Eq. 9

Where:
- $ACFW_{share}$ is the share, in percentage mass, of avoidable consumer food waste relative to all food waste reaching municipal waste treatment activities.

We then allocate a share of the total emissions (retrieved from FAOSTAT) from municipal waste treatment for food waste for a given country to its ACFW.

$$ACFW_{municipal\ waste\ treatment\ emissions} = ACFW_{share} * FW_{total\ emissions}$$

Eq. 10

Where:
- $ACFW_{municipal\ waste\ treatment\ emissions}$ is the GHG emissions associated with the waste treatment of ACFW in a given country
- $FW_{total\ emissions}$ is the total amount of GHG emissions (in tCO_2eq) from the municipal waste treatment of food waste.

The potential GHG emissions reduction from halving ACFW in each country are then computed by halving the total emissions from ACFW of the country. While we computed the avoided production and end-of-life GHG emissions since we were particularly interested on the impacts of ACFW in places of production and waste, we did not include other sectors such as transportation, processing, wholesale, retail, hotel and restaurant food emissions. The environmental benefits, considering the full life-cycle of consumer food waste could therefore be expected to be larger than the figures presented in this study.

PNV and Carbon sequestration opportunities

Regarding the carbon sequestration benefits, we adopt Sun et al.'s approach (2022) where agricultural production is mapped using SPAM to spatially explicit cropland and pastureland, which we linked to the latest harmonized global AGBC and BGBC map (Spawn et al.[19]); a SOC stock map of the top 100 cm (De Sousa et al.[20]); and a PNV map with AGBC, BGBC and SOC (Erb et al.[22]; Sanderman et al. 2017[23]). For both AGBC and BGBC, we allocated them into grid cells based on the spatial distribution of the 29 crops in SPAM and the fodder crop map in EarthStat.

$$AGBC = y\omega\,(\,0.451h^{-1} + 1.025c - 0.451)$$

Eq. 11

$$BGBC = 0.451yrh^{-1}$$

Eq. 12

Where:

- y is the production of a specific crop or fodder item
- ω is the dry-matter fraction of its harvested biomass,
- h is its harvest index (fraction of total AGBC collected at harvest),
- c is the carbon-content fraction of its harvested dry mass
- r is the root-to-shoot ratio of the crop

We determine the resulting carbon sequestration potential as the difference between the carbon stock of PNV and that of current use, using the work of Erb et al.[21] for the AGBC and BGBC, and Sanderman et al.[22], for the SOC. For the latter, we follow Sun et al.'s approach of a one-time 'committed' mass of carbon that is sequestered over an unspecified period after restoration is initiated (in practice on the order of 40-60 years). For further details on the carbon sequestration model, Sun et al., provided a detailed account of the methodologies, datasets, and assumption. Finally, we estimated the amounts of potential carbon sequestered due to sparing 50% of the land that were dedicated to produce ACFW.

Supplementary Sensitivity Analysis Results

Our study integrates multiple datasets and modelling approaches, introducing uncertainties in both data and modelling techniques. The estimation of the impacts of specific food commodities in individual nations should be interpreted cautiously due to uncertainties surrounding food waste data, trade flows, and environmental impact assessments. While we attempted to mitigate these uncertainties through rigorous data harmonization and sensitivity analyses, inherent limitations persist.

Specifically, regarding uncertainties surrounding food waste estimates, our study relies on global food waste estimates primarily derived from the Food and Agriculture Organization (FAO) report published in 2011. However, the temporal coverage and methodological variations in food waste studies pose challenges in quantifying precise uncertainty ranges[23]. For instance, a preliminary uncertainty analysis conducted by Coudard et al.[11] considered variations in food waste estimates over time, showing how estimates for specific commodities may fluctuate across different time intervals.

In this study, we conducted a broader sensitivity analysis on the data used in the ACFW dataset. UNEP Food Index annexes provided a list of all countries, each with a confidence label from *High confidence* to *Very low confidence*. Confidence ranges were then built following the report's suggestions - by attributing the confidence level to a specific confidence range for each country. The confidence range corresponding to each confidence level can be found below.

Table S17: Confidence level and confidence ranges adapted from UNEP Food Waste Index, 2022

**Confidence in estimate (UNEP, 2022)**	**Min range**	**Max range**
High confidence	-15%	+15%
Medium confidence	-35%	+35%
Low Confidence	-50%	+50%
Very Low Confidence	-60%	+60%

We used the suggested min and max confidence ranges and applied them to our AFCW dataset and further integrated the results into our MRIO model. The figures and maps below (Fig. S12-S.26) illustrate the results of this sensitivity analysis.

The sensitivity analysis results for global harvested land vary from 127 Mha to 270 Mha. The blue water consumption results vary from 38 Gm^3 to 82 Gm^3. The GHG results show production emissions varying from 254 $MtCO_2eq$ to 540 $MtCO_2eq$ while the waste treatment emissions varied from 335 $MtCO_2eq$ to 530 $MtCO_2eq$, globally. The carbon sequestration potential from halving AFCW vary from 17 $GtCO_2eq$ to 35 $GtCO_2eq$.

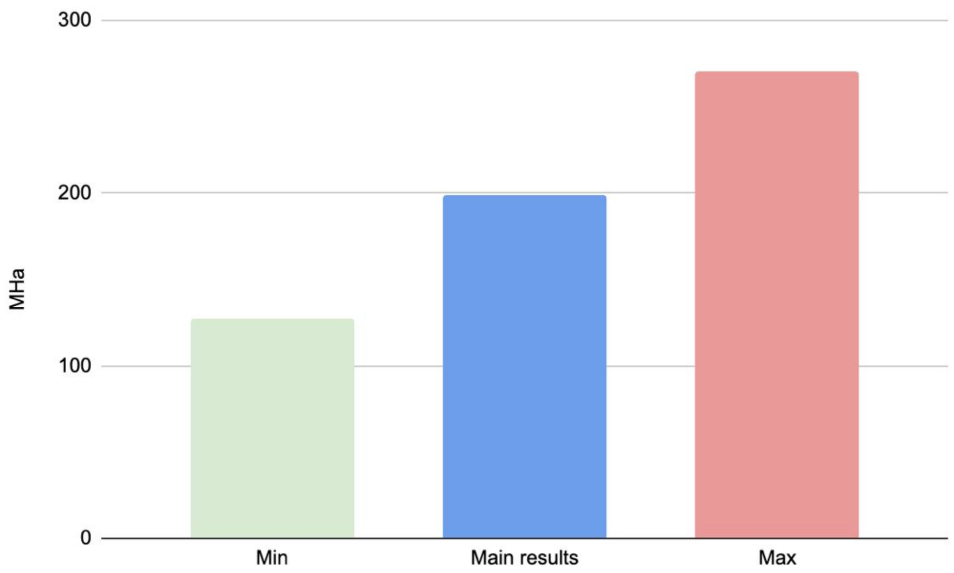

Fig. S12 – Sensitivity Analysis Results for Harvested Land

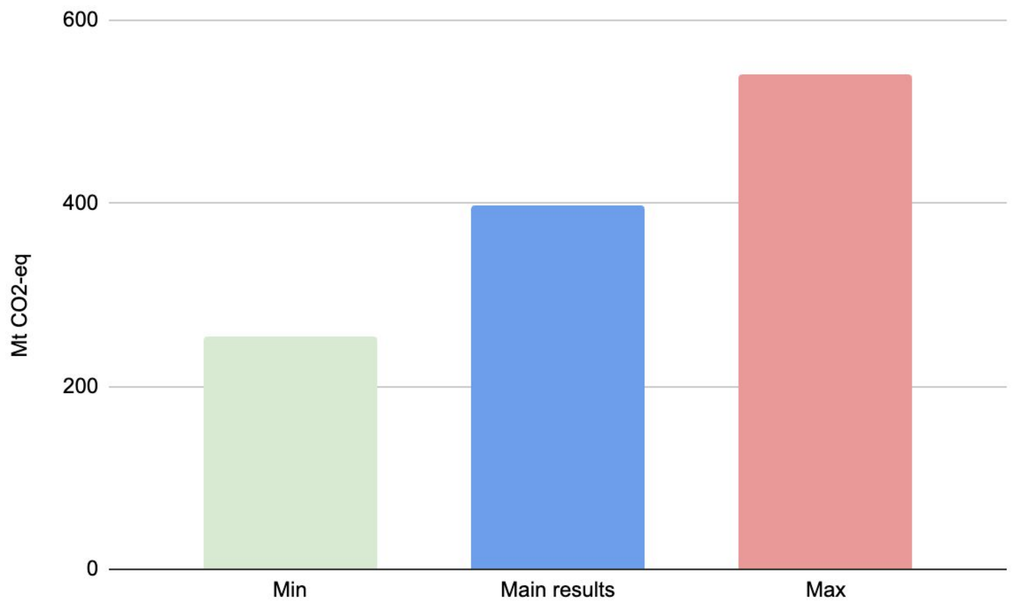

Fig. S13 – Sensitivity Analysis Results for Production-Based GHG emissions

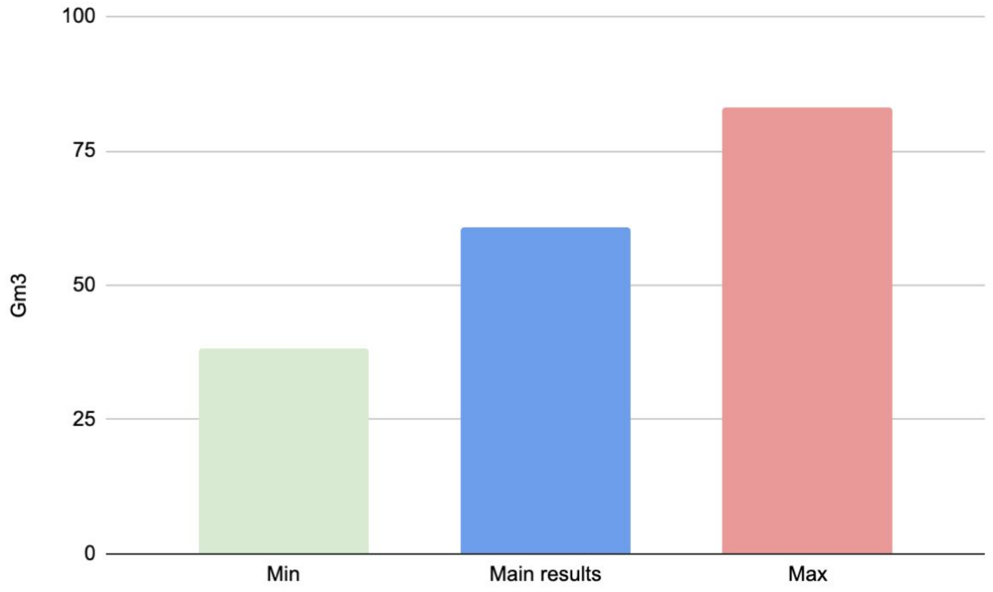

Fig. S14 – Sensitivity Analysis Results for Blue Water Consumption

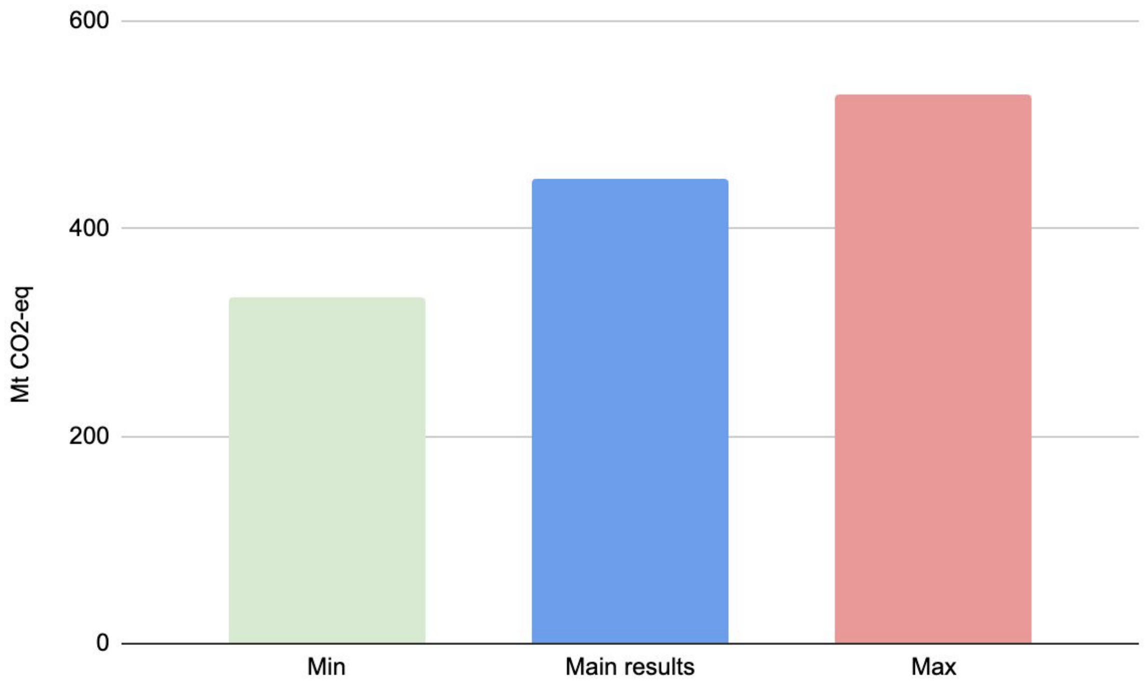

Fig. S15 – Sensitivity Analysis Results for End-of-Life GHG Analysis

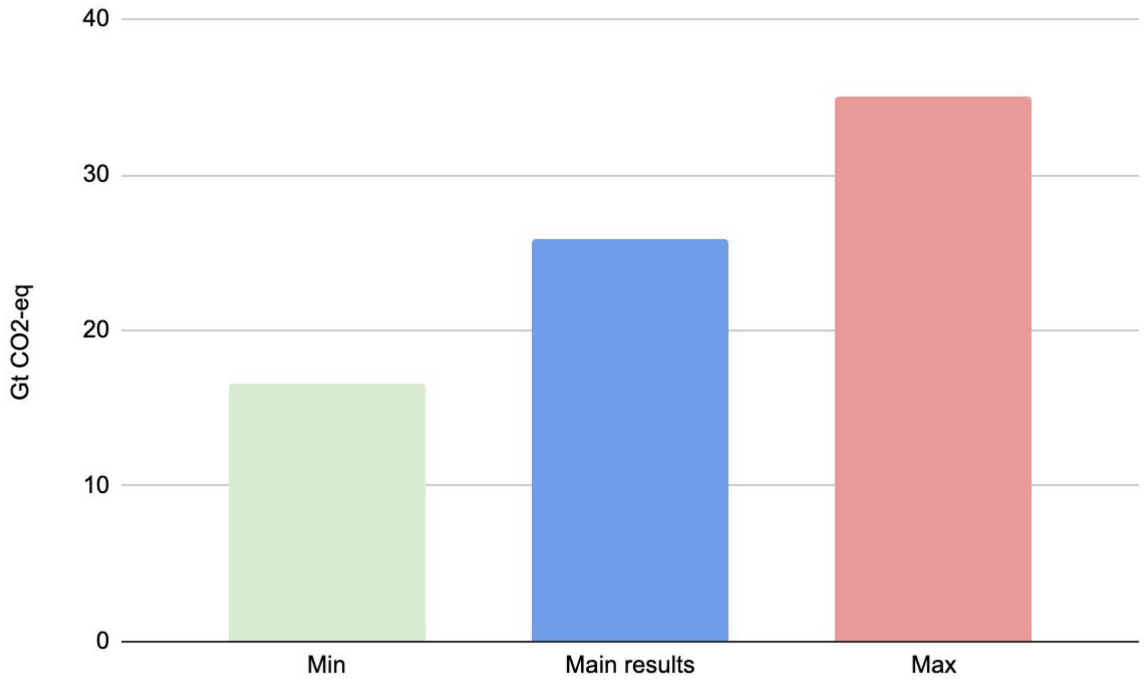

Fig. S16 – Sensitivity Analysis Results for carbon sequestration potential (halving scenario)

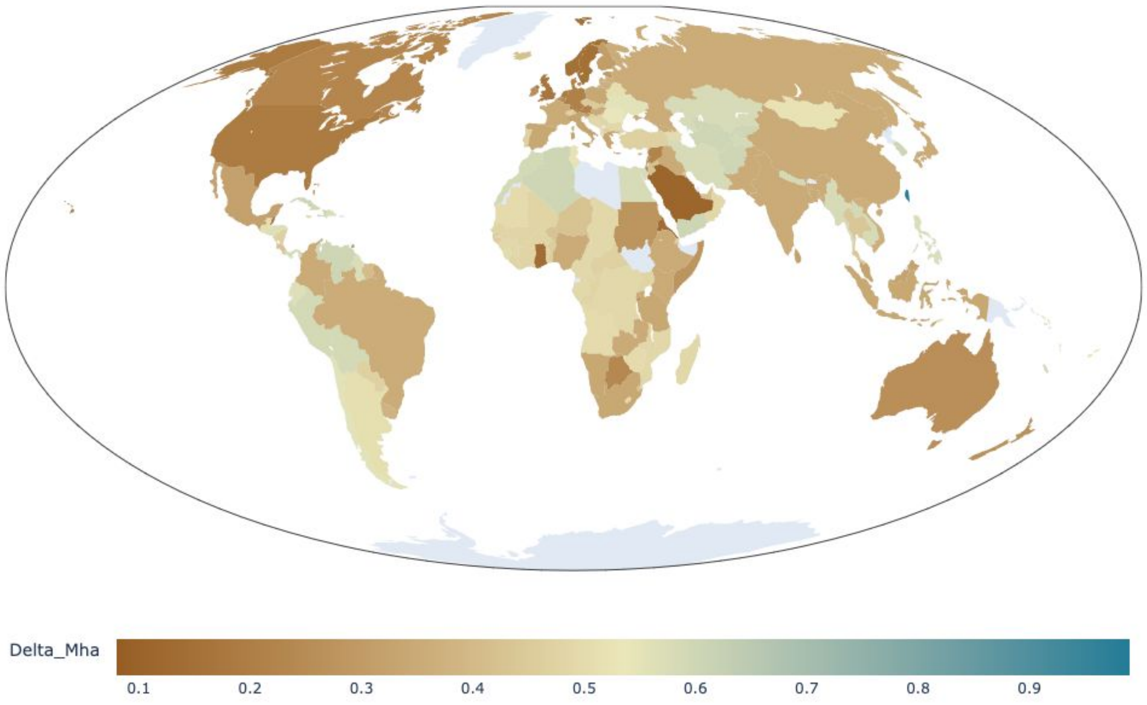

Fig. S17 – Sensitivity Analysis Delta Results for each country for the harvested land with the lower bound of the uncertainty range.

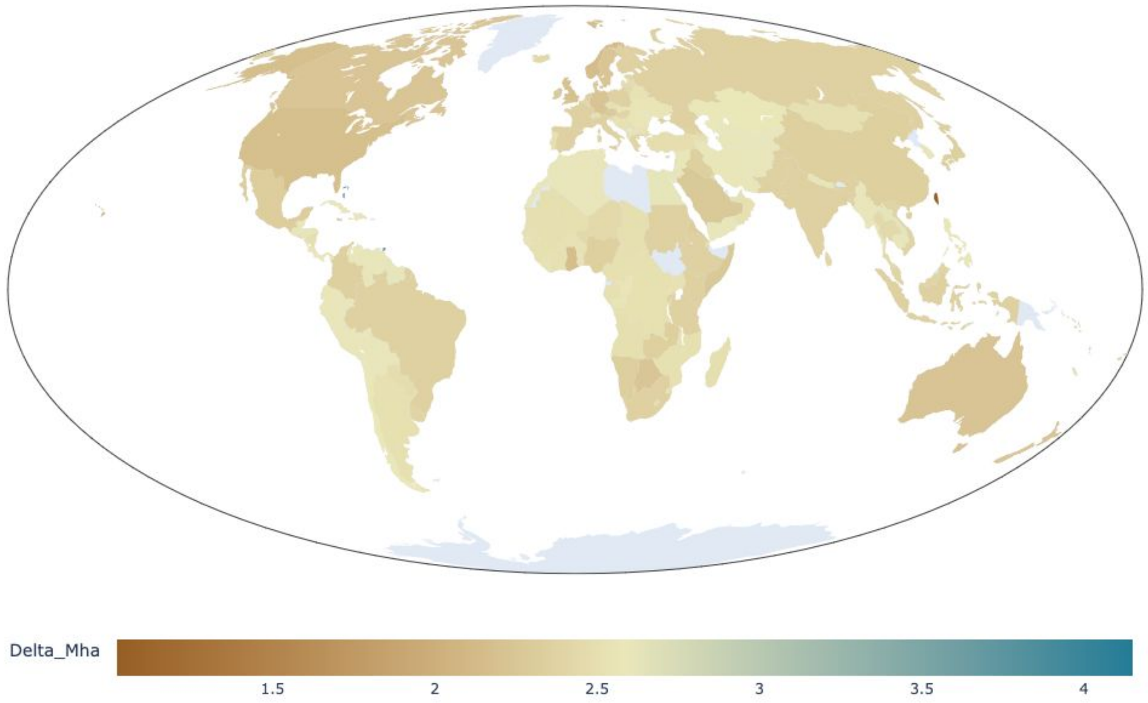

Fig. S18 – Sensitivity Analysis Delta Results for each country for the harvested land with the higher bound of the uncertainty range.

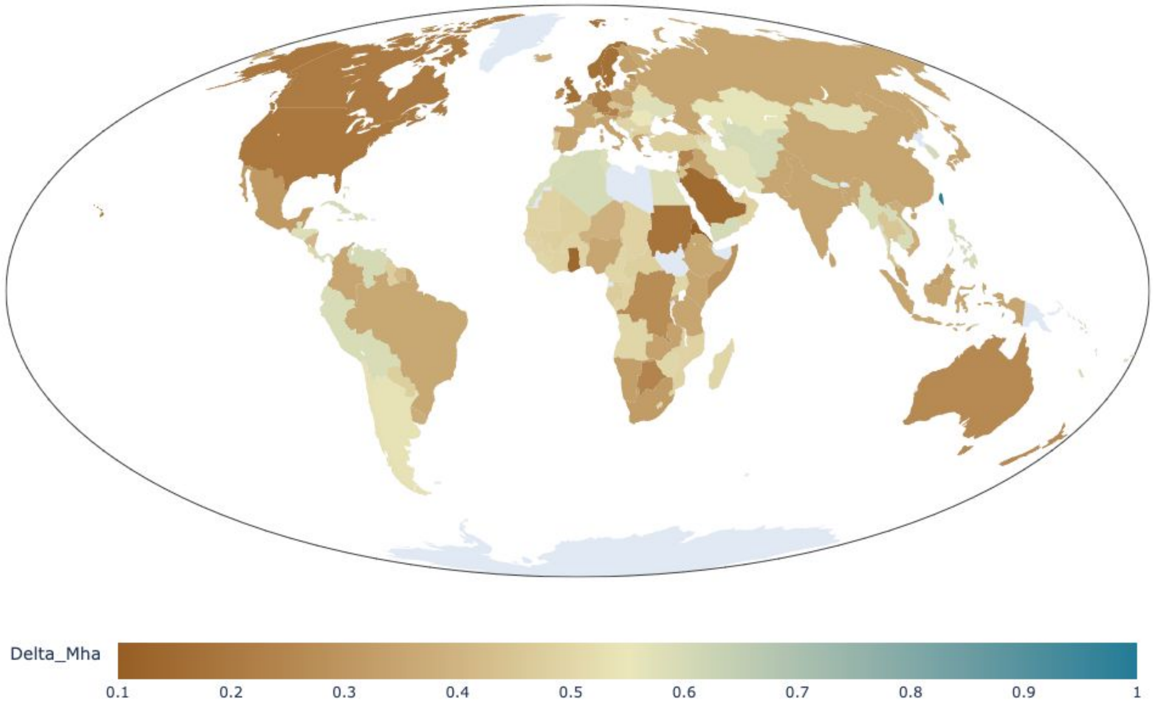

Fig. S19 – Sensitivity Analysis Delta Results for each country for the production-based GHG emissions with the lower bound of the uncertainty range.

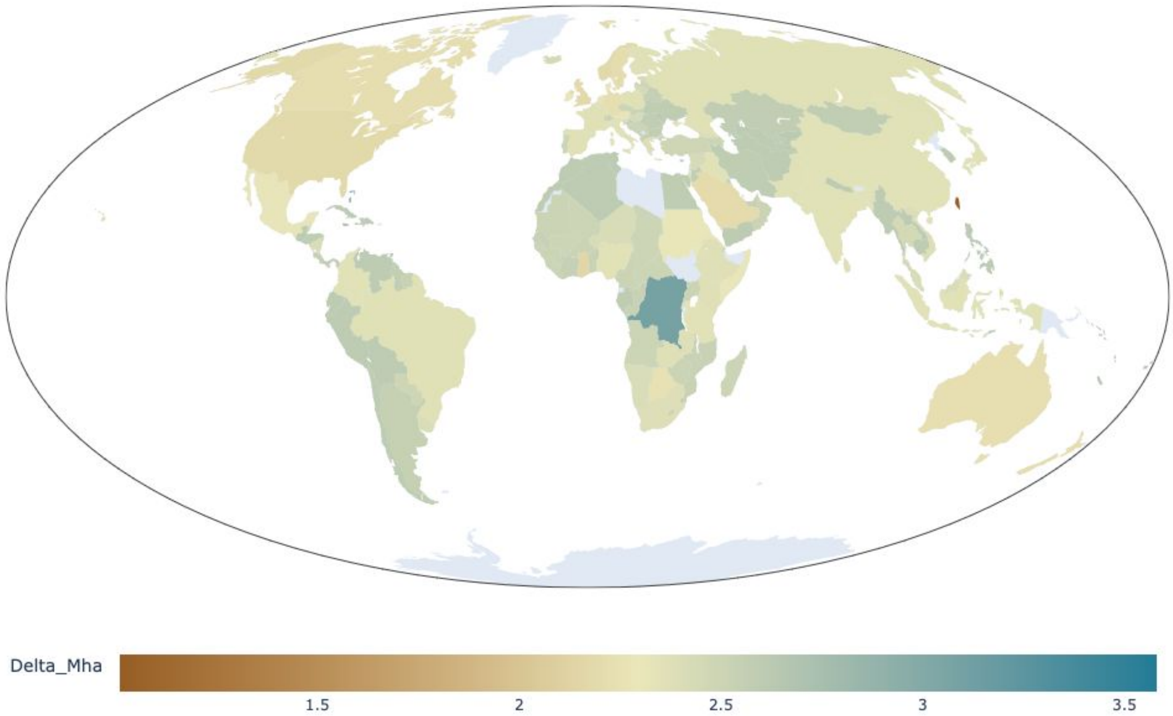

Fig. S20 – Sensitivity Analysis Delta Results for each country for the production-based GHG emissions with the higher uncertainty range.

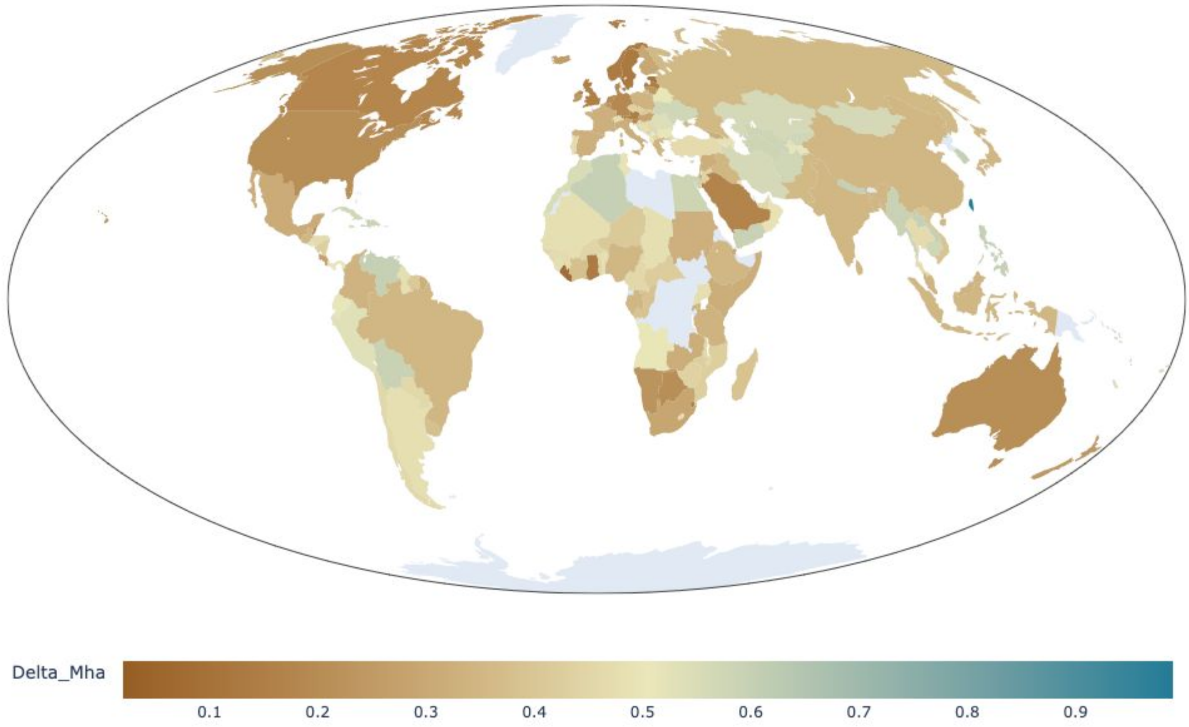

Fig. S21 – Sensitivity Analysis Delta Results for each country for the blue water with the lower bound of the uncertainty range.

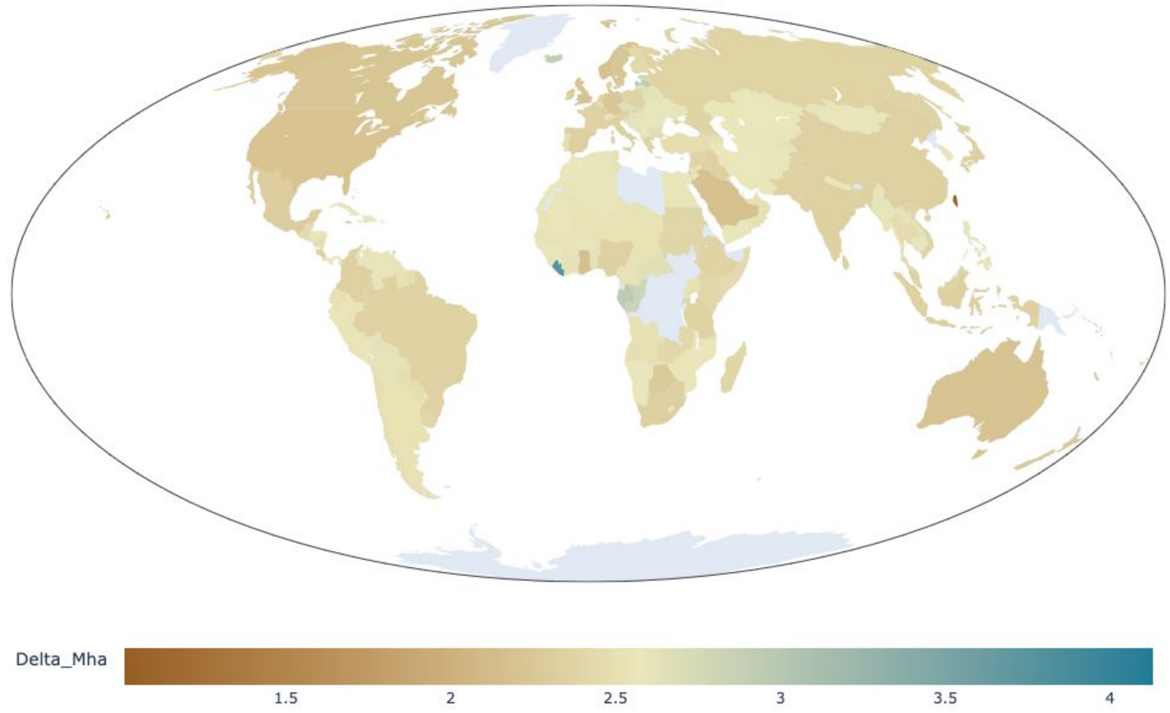

Fig. S22 – Sensitivity Analysis Delta Results for each country for the blue water land with the higher uncertainty range.

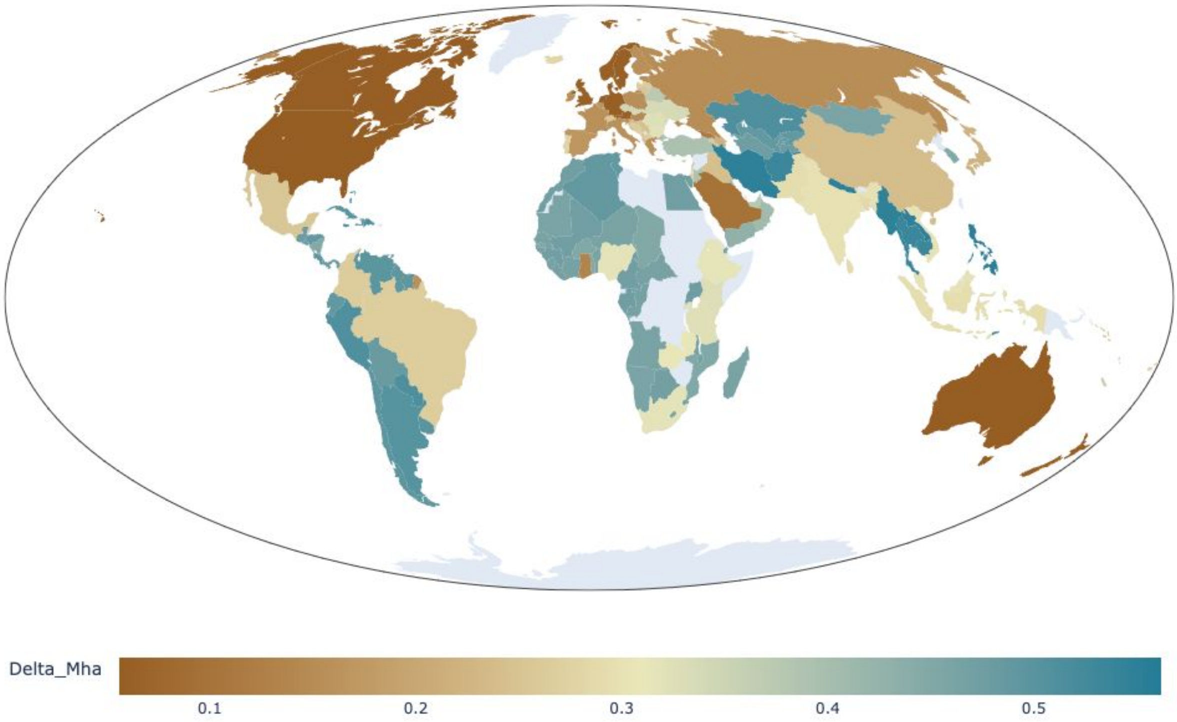

Fig. S23 – Sensitivity Analysis Delta Results for each country for the End-of-life based GHG emissions with the lower bound of the uncertainty range.

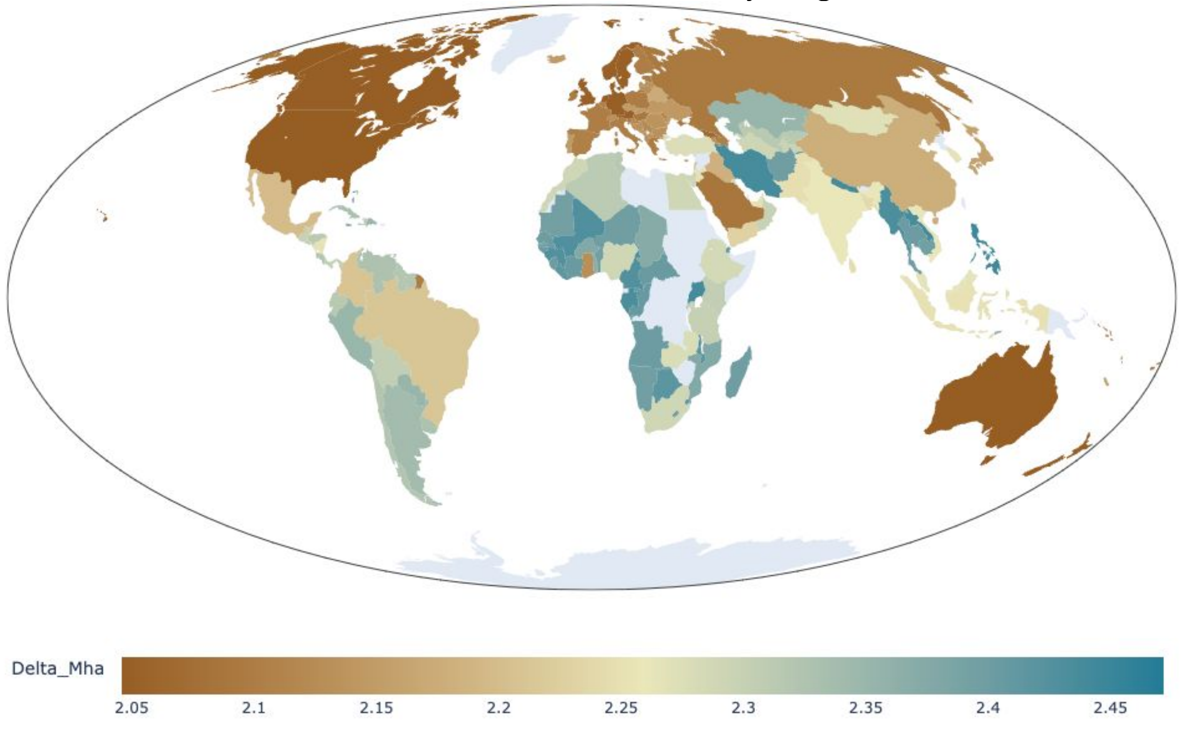

Fig. S24 – Sensitivity Analysis Delta Results for each country for the End-of-life based GHG emissions with the higher bound of the uncertainty range.

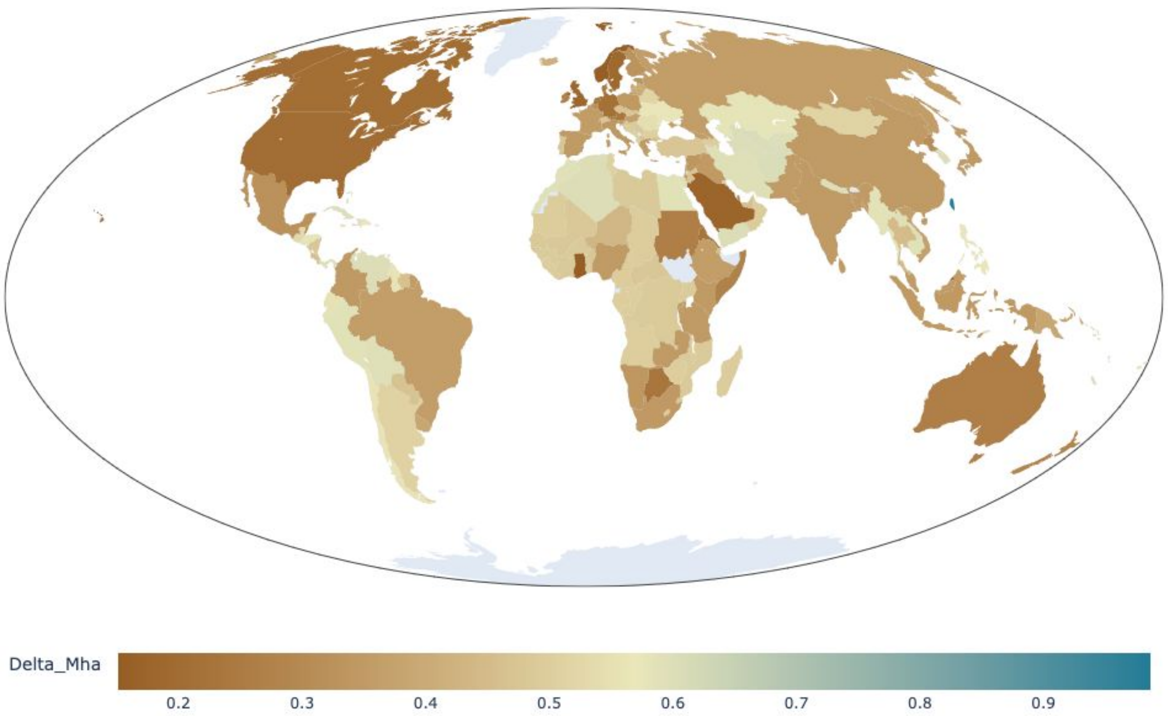

Fig. S25 – Sensitivity Analysis Delta Results for each country for the carbon sequestration potential with the lower bound of the uncertainty range.

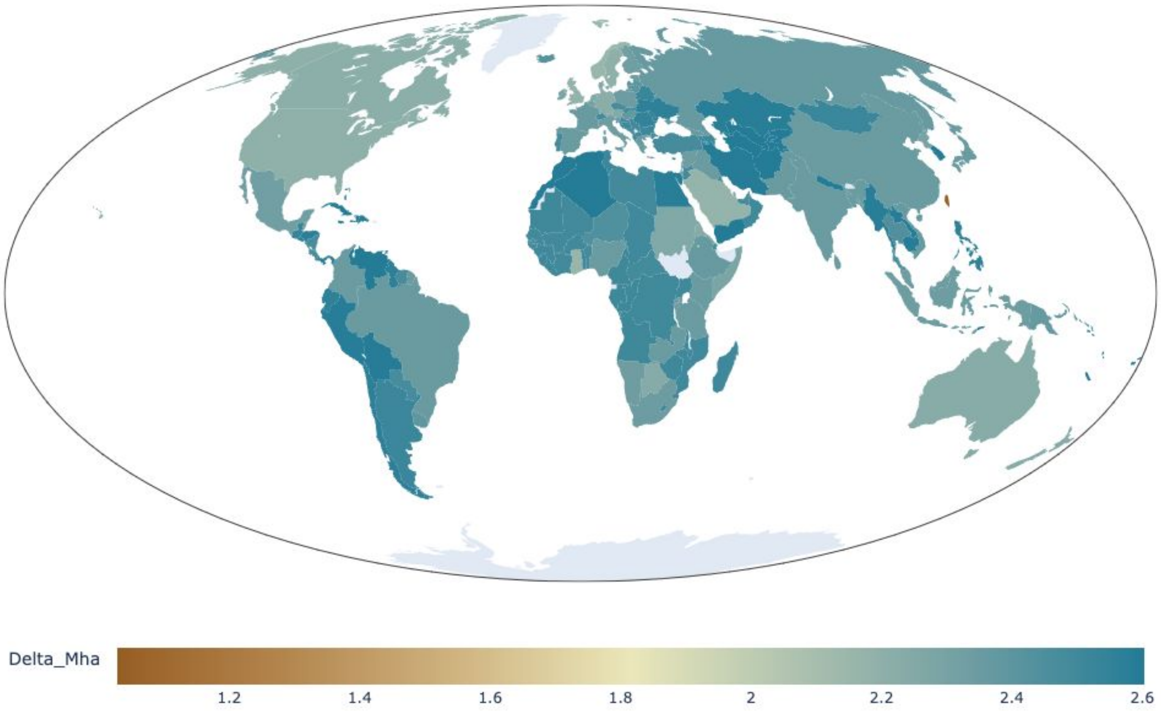

Fig. S26 – Sensitivity Analysis Delta Results for each country for the carbon sequestration potential with the higher uncertainty range.

Carbon sequestration potential

We estimated the amounts of potential carbon sequestered due to sparing the land that were dedicated to produce food that not eaten at the consumer stage following Sun et al.'s approach. Significant uncertainties, associated with biomass carbon estimates and soil organic carbon predictions remain in this aspect, underscoring the complexity of accurately estimating carbon sequestration potential. For these uncertainties, please refer to Sun et al. as they have addressed them through their own sensitivity analyses focusing on factors such as aboveground biomass carbon, belowground biomass carbon, and soil organic carbon (SOC). For biomass carbon, Sun et al. used the uncertainty dataset from Spawn et al.[37], which was then used in the model framework to assess overall uncertainties. Future revisions to datasets, such as FABIO or SPAM, may also introduce additional uncertainties to our findings. Despite efforts to quantify and account for these uncertainties, ongoing advancements in data availability and modelling techniques may necessitate continual reassessment of our findings. Further uncertainty analysis is described in Sun et al.[11].

References

[1] Coudard, A., Corbin, E., Koning, de J., Tukker, A., Mogollón, J.M. Global water and energy losses from consumer avoidable food waste. *Journal of Cleaner Production*. Volume 326. (2021). https://doi.org/10.1016/j.jclepro.2021.129342.
[2] Food and Agriculture Organization. FAOSTAT Statistical Database. 2023. License: CC BY-NC-SA 3.0 IGO.
[3]Vanham, D., Mak, T. N., & Gawlik, B. M.. Urban food consumption and associated water resources: The example of Dutch cities. Science of The Total Environment, 565, 232–239. https://doi.org/10.1016/J.SCITOTENV.2016.04.172 (2016)
[4] Food and Agriculture Organization. Technical Conversion Factors for Agricultural Commodities. https://www.fao.org/fileadmin/templates/ess/documents/methodology/tcf.pdf (2003)
[5] Bruckner M., Wood R., Moran D., Kuschnig N., Wieland H., Maus V., Börner J. FABIO—The Construction of the Food and Agriculture Biomass Input–Output Model. Environmental Science & Technology 2019 53 (19), 11302-11312 DOI: 10.1021/acs.est.9b03554 (2019)
[6] Food and Agriculture Organization of the United Nations. Global Food Losses and Food Waste: Extent, Causes and Prevention. Rome: FAO. (2011). http:// www.fao.org/3/i2697e/i2697e.pdf
[7] De Laurentiis, V., Corrado, S. & Sala, S.. Quantifying household waste of fresh fruit and vegetables in the EU. Waste Management 77, 238–251. https://doi.org/10.1016/j.wasman.2018.04.001 (2018)
[8]WRAP. Household food and drink waste: A product focus – Final Report. 978-1-84405-469-5 (2014)
[9] John-Jaja, S. A., Udoh, U. H. & Nwokolo, S. C.. Repeatability estimates of egg weight and egg-shell weight under various production periods for Bovan Nera Black laying chicken. Beni-Suef University Journal of Basic and Applied Sciences (2016). doi:10.1016/j.bjbas.2016.11.001
[10] Xue, L., Liu, G., Parfitt, J., Liu, X., Van Herpen, E., Stenmarck, Å., Cheng, S. Missing Food, Missing Data? A Critical Review of Global Food Losses and Food Waste Data. Environmental Science & Technology, 51(12), 6618–6633. doi:10.1021/acs.est.7b00401 (2017)
[11] Sun, Z., Scherer, L., Tukker, A., Spawn-Lee, S., Bruckner, M., Gibbs, H., K., Behrens, P. Dietary change in high-income nations alone can lead to substantial double climate dividend. Nat Food 3, 29–37. (2022). https://doi.org/10.1038/s43016-021-00431-5
[12] International Food Policy Research Institute Global Spatially-Disaggregated Crop Production Statistics Data for 2010 version 2.0. *Harvard Dataverse.* (2019). https://doi.org/10.7910/DVN/PRFF8V.
[13] Ramankutty, N., Evan, A. T., Monfreda, C. & Foley, J. A. Farming the planet: 1. geographic distribution of global agricultural lands in the year 2000. *Glob. Biogeochem. Cycles*. (2008) https://doi.org/10.1029/2007GB002952
[14] IPCC, 2014: Climate Change 2014: Synthesis Report. Contribution of Working Groups I, II and III to the Fifth Assessment Report of the Intergovernmental Panel on Climate Change [Core Writing Team, R.K. Pachauri and L.A. Meyer (eds.)]. IPCC, Geneva, Switzerland, 151 pp.
[15] Mekonnen MM., Gerbens-Leenes W. The Water Footprint of Global Food Production. *Water*. (2020). 12(10):2696. https://doi.org/10.3390/w12102696
[16] FAO. Water Use of Livestock Production Systems and Supply Chains—Guidelines for Assessment (2018).
[17] Crippa, M., Solazzo, E., Guizzardi, D., Monforti-Ferrario, F., Tubiello, F.N., Leip, A. Food systems are responsible for a third of global anthropogenic GHG emissions. Nat. Food 2, 198–209. (2021)
[18] World Bank. Methane-Reducing and Water-Saving Paddy Rice Program for Results (Hunan). (2023). https://projects.worldbank.org/en/projects-operations/project-detail/P178796
[19] Spawn, S. A., Sullivan, C. C., Lark, T. J. & Gibbs, H. K. Harmonized global maps of above and belowground biomass carbon density in the year 2010. Sci. Data 7, 112 (2020)
Poggio, L., de Sousa, L.M., Batjes, N.H, Heuvelink, G.B.M., Kempen, B., Ribeiro, E., and Rossiter, D. SoilGrids 2.0: producing soil information for the globe with quantified spatial uncertainty. *Soil* 7, 217–240 (2021).
[21] Erb, K.-H., Kastner, T., Plutzar, C., Bais, A. L. S., Carvalhais, N., Fetzel, T., Gingrich, S., Haberl, H., Lauk, C., Niedertscheider, M., Pongratz, J., Thurner, M., Luyssaert, S. Unexpectedly large impact of forest management and grazing on global vegetation biomass. Nature 553, 73–76. (2018)
[22] Sanderman J, Hengl T and Fiske G J 2017 Soil carbon debt of 12 000 years of human land use. *Proc. Natl Acad. Sci*. 114 9575–80
